# Short-term tamoxifen administration improves hepatic steatosis and glucose intolerance through JNK/MAPK in mice

**DOI:** 10.1038/s41392-022-01299-y

**Published:** 2023-03-03

**Authors:** Zhiqiang Fang, Hao Xu, Juanli Duan, Bai Ruan, Jingjing Liu, Ping Song, Jian Ding, Chen Xu, Zhiwen Li, Kefeng Dou, Lin Wang

**Affiliations:** 1 Department of Hepatobiliary Surgery, Xi-Jing Hospital, Fourth Military Medical University, Xi’an, 710032 China; 2 Center of Clinical Aerospace Medicine & Department of Aviation Medicine, Fourth Military Medical University, Xi’an, 710032 China

**Keywords:** Translational research, Drug development

## Abstract

Nonalcoholic fatty liver disease (NAFLD) which is a leading cause of chronic liver diseases lacks effective treatment. Tamoxifen has been proven to be the first-line chemotherapy for several solid tumors in clinics, however, its therapeutic role in NAFLD has never been elucidated before. In vitro experiments, tamoxifen protected hepatocytes against sodium palmitate-induced lipotoxicity. In male and female mice fed with normal diets, continuous tamoxifen administration inhibited lipid accumulation in liver, and improved glucose and insulin intolerance. Short-term tamoxifen administration largely improved hepatic steatosis and insulin resistance, however, the phenotypes manifesting inflammation and fibrosis remained unchanged in abovementioned models. In addition, mRNA expressions of genes related to lipogenesis, inflammation, and fibrosis were downregulated by tamoxifen treatment. Moreover, the therapeutic effect of tamoxifen on NAFLD was not gender or ER dependent, as male and female mice with metabolic disorders shared no difference in response to tamoxifen and ER antagonist (fulvestrant) did not abolish its therapeutic effect as well. Mechanistically, RNA sequence of hepatocytes isolated from fatty liver revealed that JNK/MAPK signaling pathway was inactivated by tamoxifen. Pharmacological JNK activator (anisomycin) partially deprived the therapeutic role of tamoxifen in treating hepatic steatosis, proving tamoxifen improved NAFLD in a JNK/MAPK signaling-dependent manner.

## Introduction

Nonalcoholic fatty liver disease (NAFLD) which is a metabolic-associated chronic liver disease,^1,2^ has become the leading cause of hepatocellular carcinoma^3^ and the leading reason for liver transplantation in western countries.^4^ In terms of pathological characteristics, NAFLD can be categorized as nonalcoholic fatty liver and nonalcoholic steatohepatitis (NASH) which is prone to end-stage liver diseases.^5^ The pathogenesis of NAFLD is complex and tightly connected with obesity, type 2 diabetes mellitus, hyperlipidemia, and metabolic syndrome.^6^ Although the disease burden is increasing, no FDA-approved drug has been found to effectively treat NAFLD or NASH so far.

Tamoxifen, synthesized in 1966, is a selective estrogen receptor (ER) modulator (SERM).^7^ Since there are multiple target tissues for estrogen, such as endometrium, cardiovascular system, bone, brain, and liver,^8^ off-target effects may occur following tamoxifen administration. Tamoxifen has been used for the treatment of breast cancer for 50 years and the first case was reported in 1971.^9^ Growing evidence unveiled long-term tamoxifen treatment in breast cancer patients led to metabolic disorders. In 1995, Pratt et al. first discovered tamoxifen treatment resulted in steatohepatitis.^10^ Afterwards, Van Hoof, Ogawa, and Cai et al. reported the similar clinical phenomenon.^11–13^ However, a clinical trial subsequently demonstrated that tamoxifen only increased the risk of NASH in overweight and obese women.^14^ In addition, a few in vitro and in vivo experiments proved that tamoxifen promoted lipid accumulation in hepatocytes and enhanced fatty acid biosynthesis.^15–19^ However, some other findings suggested that tamoxifen protected hepatocytes against lipotoxicity and steatosis.^20,21^ Therefore, the effect of tamoxifen on lipid metabolism seems to be controversial.

Apart from cancer treatment, tamoxifen is frequently used to generate genetic mutations in mice by inducing Cre/loxp recombination system. The CreER/loxp system which is widely used in transgenic mice can induce somatic mutations at a chosen time and/or in a specific tissue.^22^ Through fusing Cre recombinase with a mutated ligand-binding domain of ER, the Cre recombinase can be activated by tamoxifen instead of estradiol.^23^ The route of tamoxifen induction varies from different laboratories. Wang et al. administrated tamoxifen orally to adult mice at 100 mg/kg for 4 consecutive days to induce Cre recombination,^24^ while in O’Shea’s publication, tamoxifen was intraperitoneally injected at 50 mg/kg per day for 5 consecutive days.^25^ Unavoidably, some off-target effects of tamoxifen induction were reported in retina and skeleton.^26,27^ Occasionally, we found that following a normal routine of tamoxifen induction, lipid accumulation and hepatic steatosis were prominently reduced in both normal and NAFLD-established mice, which aroused our great interest. It seems that the off-target effect of tamoxifen may probably be protective to lipid metabolism.

In this article, we investigated the role of short-term tamoxifen administration in NAFLD mouse models and determined JNK/MAPK signaling pathway was involved in tamoxifen-driven treatment. Also, we discussed the safety and potential application of tamoxifen in the treatment on NAFLD.

## Results

### Tamoxifen protects hepatocytes against lipotoxicity in vitro

**Fig. 1:**
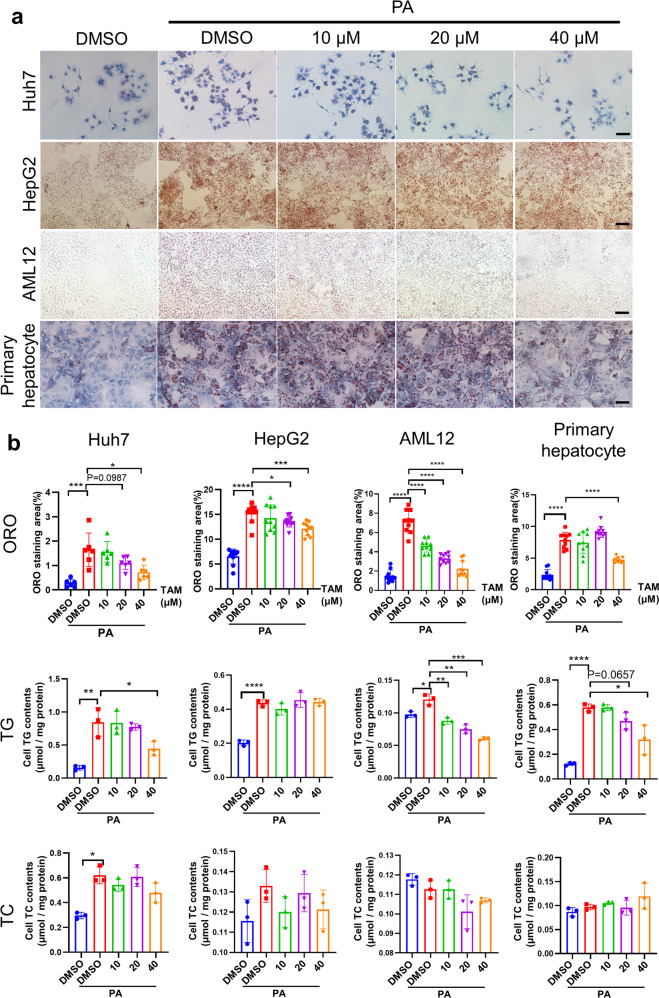
Tamoxifen decreased hepatocyte lipotoxicity in vitro. **a** Huh7, HepG2, AML12 cells, and primary hepatocytes were seeded in six-well plates. After 12 h, 0.3 mM sodium palmitate was added to the medium and after 36 h, DMSO/tamoxifen (10, 20, 40 μM) was added. After 36 h, cells were stained with ORO, pictured using an inverted phase contrast microscope, and quantified by ORO staining areas. Scale bar: 100 μm. **b** Statistical analysis of ORO staining. For cellular TC and TG test, cells were digested by trypsin, washed with PBS and collected using centrifuge. The following steps were performed according to the manufacturer’s protocols. All experiments were repeated at least three biological times. Bars = means ± SD; *n* = 3–10; *P < 0.05; **P < 0.01; ***P < 0.001; ****P < 0.0001

To explore the impact of tamoxifen on hepatocytes in vitro, we utilized primary hepatocytes, two human hepatocyte cell lines—Huh7 and HepG2 and a mouse hepatocyte cell line—AML12. To induce lipotoxicity, cells were exposed to 0.3 mM sodium palmitate for 36 h. For treatment, different concentrations of tamoxifen (10, 20, 40 μM) were added into cell medium for another 36 h. As shown by Oil-red O (ORO) staining (Fig. 1a, b), in primary hepatocytes and all three cell lines, sodium palmitate-triggered lipid accumulation could be notably reduced by tamoxifen in a dose-dependent manner. However, tamoxifen treatment did not decrease cellular TC contents in all these cells and did not change TG contents in HepG2 cell line. To investigate whether tamoxifen increased cytotoxicity, we performed trypan blue staining in Huh7 and AML12 cell lines treated with PA or PA plus different concentrations of TAM to examine cell viability and found that tamoxifen treatment did not increase cytotoxicity (Supplementary Fig. S1a, b). To investigate the role of tamoxifen on lipid metabolism, we performed RT-qPCR experiment and found that tamoxifen suppressed expressions of genes related to lipid uptake (FATP2, FATP5, and CD36), de novo lipogenesis (SREBP1c, FASN, SCD1, and ACC1), fatty acid oxidation (PPARα, CPT1α, and ACOX1) and TG export (APOB and MTTP) in a dose-dependent manner (Supplementary Fig. S1c). These data collectively indicated that tamoxifen could effectively protect hepatocytes from lipotoxicity in vitro without increasing cytotoxicity.

### Continuous tamoxifen administration does not worsen liver function in normal mice

**Fig. 2:**
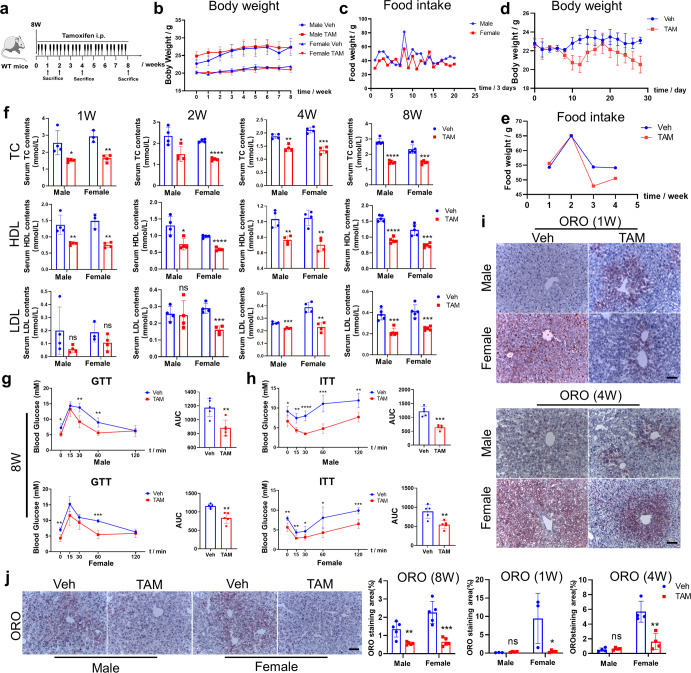
Tamoxifen inhibited lipid accumulation in mice fed with normal diet. **a** Dosing scheme of tamoxifen on male and female C57BL/6 mice fed with normal diets. Dose of tamoxifen: 100 mg/kg. **b** Since initiating tamoxifen administration, mice were pair-fed and body weight was recorded weekly until mice were sacrificed. **c** Food consumption weight per cage was recorded when mice were administrated with tamoxifen. **d** Since initiating tamoxifen administration, mice were free-fed and body weight was recorded every other day for 4 weeks. **e** Food consumption weight per cage was recorded weekly when free-fed mice were administrated with tamoxifen. **f** Serum TC, HDL-C and LDL-C analysis of male and female mice fed with normal diets and administrated with vehicle/tamoxifen for 1, 2, 4, 8 weeks, respectively. **g** GTT test was performed on male and female mice administrated with tamoxifen or vehicle for 8 weeks and area under curve (AUC) was calculated and compared. **h** ITT test was performed on male and female mice administrated with tamoxifen or vehicle for 8 weeks and area under curve (AUC) was calculated and compared. **i** Frozen liver sections from male and female mice administrated with tamoxifen or vehicle for 1 and 4 weeks was performed ORO staining and ORO staining area was quantitatively compared. Scale bar:100 μm. **j** Frozen liver sections from male and female mice administrated with tamoxifen or vehicle for 8 weeks were performed ORO staining and ORO staining area was quantitatively compared. Scale bar:100 μm. Bars = means ± SD; *n* = 3– 5; ns, no significance; *P < 0.05; **P < 0.01; ***P < 0.001; ****P < 0.0001

We then evaluated the role of continuous injection of tamoxifen in normal mice. To investigate whether tamoxifen decreases food intake and body weight due to appetite suppression, mice with free access to normal food and water were administrated with 100 mg/kg tamoxifen intraperitoneally every other day for 4 weeks. With the prolongation of tamoxifen treatment, the food intake and body weight decreased in tamoxifen-treated group (Fig. 2d, e). To exclude the effect of appetite suppression, male and female C57BL/6 mice were pair-fed with normal diet and administrated with tamoxifen intraperitoneally for 1, 2, 4, and 8 weeks, respectively (Fig. 2a). Continuous tamoxifen administration for 8 weeks made no change to the body weight either in male or in female mice (Fig. 2b), indicating pair-feeding largely offset the potential side-effect of tamoxifen on appetite suppression. As shown in Fig. 2c, no decline of food intake was observed as well. Then, ALT and AST tests confirmed that continuous tamoxifen injection did not worsen liver function (Supplementary Fig. S2a). On weeks 4 and 8, the serum level of ALT even decreased a little bit by using tamoxifen, which caused no hepatotoxicity.

### Tamoxifen inhibits lipid accumulation in mice fed with normal diet

Afterwards, we detected lipid accumulation in blood. No significant difference was found in serum TG levels between the tamoxifen-treated group and the control (Supplementary Fig. S2a). However, serum TC, HDL-C, and LDL-C levels were distinctly lowered by tamoxifen, unveiling the potential effectiveness of tamoxifen on cholesterol metabolism (Fig. 2f). Considering that insulin resistance is a major contributor to NAFLD, we examined glucose and insulin tolerance through GTT and ITT assays. As early as one week after tamoxifen administration, male mice exhibited better glucose tolerance while female ones showed no difference (Supplementary Fig. S2b), which was consistent with the change on week 4 (Supplementary Fig. S2c). Intriguingly, following 8-week administration, both male and female mice tolerated better with glucose and insulin in tamoxifen-treated group than in control group, which were determined by GTT and ITT assays respectively (Fig. 2g, h). To investigate lipid accumulation in liver, we performed ORO staining. One week or four weeks after tamoxifen administration, less hepatic lipid deposition could only be found in female mice (Fig. 2i). However, hepatic lipid accumulation was largely reduced by tamoxifen in both male and female mice following 8 weeks (Fig. 2j). RT-qPCR analyses showed that tamoxifen only decreased the expression of SCD1 (Supplementary Fig. S2d). These results collectively suggested that continuous injection of tamoxifen within 8 weeks could effectively decrease circulating TC contents, improve glucose and insulin intolerance and inhibit lipid deposition in mice fed with normal diet.

### Tamoxifen ameliorates MCD and CDAA-induced hepatic steatosis

**Fig. 3:**
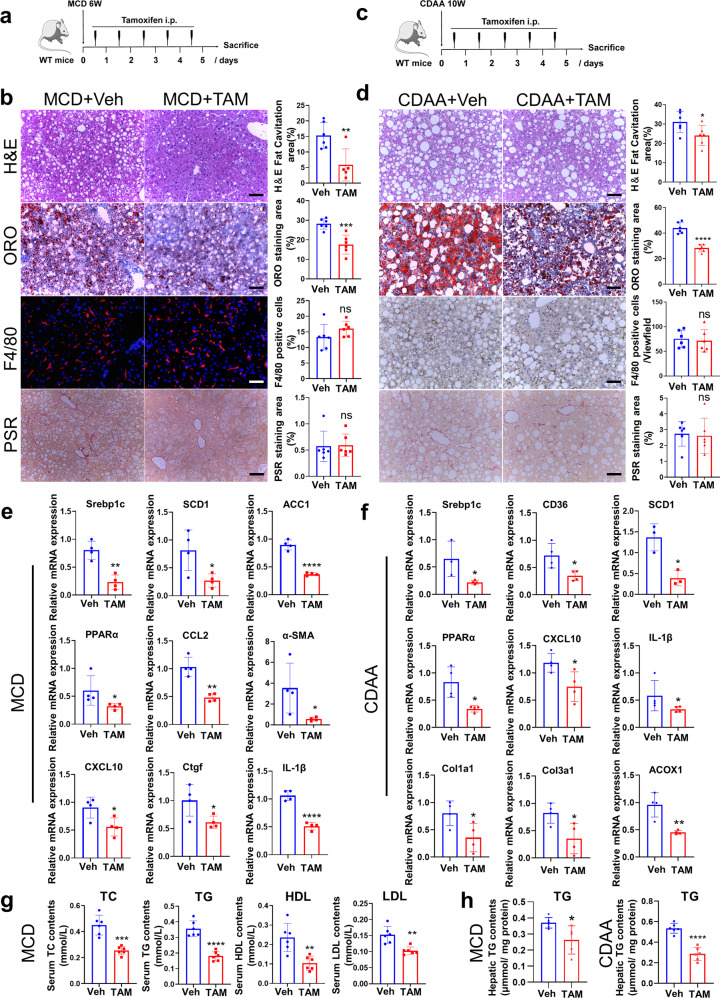
Tamoxifen alleviated hepatic steatosis in MCD and CDAA-induced models. **a** Dosing scheme of tamoxifen on male C57BL/6 mice fed with MCD diets for 6 weeks. Dose of tamoxifen: 100 mg/kg. **b** Liver sections from tamoxifen group and vehicle group in mice fed with MCD diets were performed H&E, ORO, F4/80 IF, and PSR staining. H&E fat cavitation area, ORO staining area, F4/80 positive cells percentage and PSR staining area were quantitatively compared. Scale bar: 100 μm. **c** Dosing scheme of tamoxifen on male C57BL/6 mice fed with CDAA diets for 10 weeks. Dose of tamoxifen: 100 mg/kg. **d** Liver sections from tamoxifen group and vehicle group in mice fed with CDAA diets were performed H&E, ORO, F4/80 IF, and PSR staining. H&E fat cavitation area, ORO staining area, F4/80 positive cells percentage and PSR staining area were quantitatively compared. Scale bar:100 μm. **e** RNA was extracted from liver tissues of MCD diets-induced NASH mice administrated with tamoxifen or vehicle and expression of lipogenesis, inflammation, and fibrosis-related genes was determined by RT-qPCR with β-actin as an internal control. **f** RNA was extracted from liver tissues of CDAA diets-induced NASH mice administrated with tamoxifen or vehicle and expression of lipogenesis, inflammation, and fibrosis-related genes were determined by RT-qPCR with β-actin as an internal control. **g** Serum analysis of TC, TG, HDL and LDL in MCD diets-induced mice administrated with tamoxifen or vehicle. **h** Liver tissues from MCD and CDAA-induced NASH mice administrated with tamoxifen or vehicle were harvested and TG concentrations were measured using commercial kits. Hepatic TG contents were normalized by hepatic protein levels. Bars = means ± SD; *n* = 3–6; ns, no significance; *P < 0.05; **P < 0.01; ***P < 0.001; ****P < 0.0001

To identify the potential role of tamoxifen in NASH treatment, we established MCD and CDAA diet-induced NASH mouse models. Male mice were fed with MCD diet for 6 weeks or CDAA diet for 10 weeks, and were then administrated with 100 mg/kg tamoxifen intraperitoneally for 5 consecutive days (Fig. 3a, c). In MCD diet-induced NASH mice, liver weight slightly decreased in tamoxifen-treated group while body weight and liver to body weight ratio both remained unchanged (Supplementary Fig. S3a). In mice fed with CDAA diet, no change of body weight was found before or after tamoxifen administration (Supplementary Fig. S3b). Different from MCD diets, CDAA diets didn’t cause body weight consumption (Supplementary Fig. S3c). In addition, we performed GTT experiment and found that tamoxifen partially improved glucose intolerance in CDAA diet-induced NASH mice (Supplementary Fig. S3d). ORO and H&E staining revealed that tamoxifen treatment notably decreased hepatic lipid accumulation caused by MCD and CDAA diets (Fig. 3b, d), which was confirmed by liver TG measurements (Fig. 3h). However, the number of F4/80 positive cells was not altered by tamoxifen as shown by F4/80 immunohistochemistry (IHC) and immunofluorescence (IF) staining (Fig. 3b, d). In addition, PSR staining showed that the Sirius Red positive stain was unchanged by the use of tamoxifen (Fig. 3b, d), indicating no manifestation of liver inflammation or fibrosis was observed following tamoxifen treatment.

Then we extracted RNA from liver tissues of mice fed with MCD diet. RT-qPCR analyses demonstrated that marker genes related to fatty acid synthesis (Srebp1c, SCD1, and ACC1), inflammatory response (CCL2, CXCL10, IL-1β), and fibrosis (α-SMA and Ctgf) were downregulated in tamoxifen-treated group (Fig. 3e). In CDAA diet-induced liver, genes of lipogenesis (Srebp1c, CD36, and SCD1), inflammation (CXCL10 and IL-1β) and fibrogenesis (Col1a1 and Col3a1) were also downregulated by tamoxifen and genes regarding fatty acid oxidation (PPARα and ACOX1) were reduced as well (Fig. 3f). Furthermore, tamoxifen treatment decreased serum levels of TC, TG, HDL-C and LDL-C in MCD diet-induced NASH mice (Fig. 3g). Above results proved that short-term tamoxifen treatment effectively improved hepatic steatosis in mice fed with MCD or CDAA-diet.

### Tamoxifen attenuates HFD-induced hepatic steatosis and glucose intolerance

**Fig. 4:**
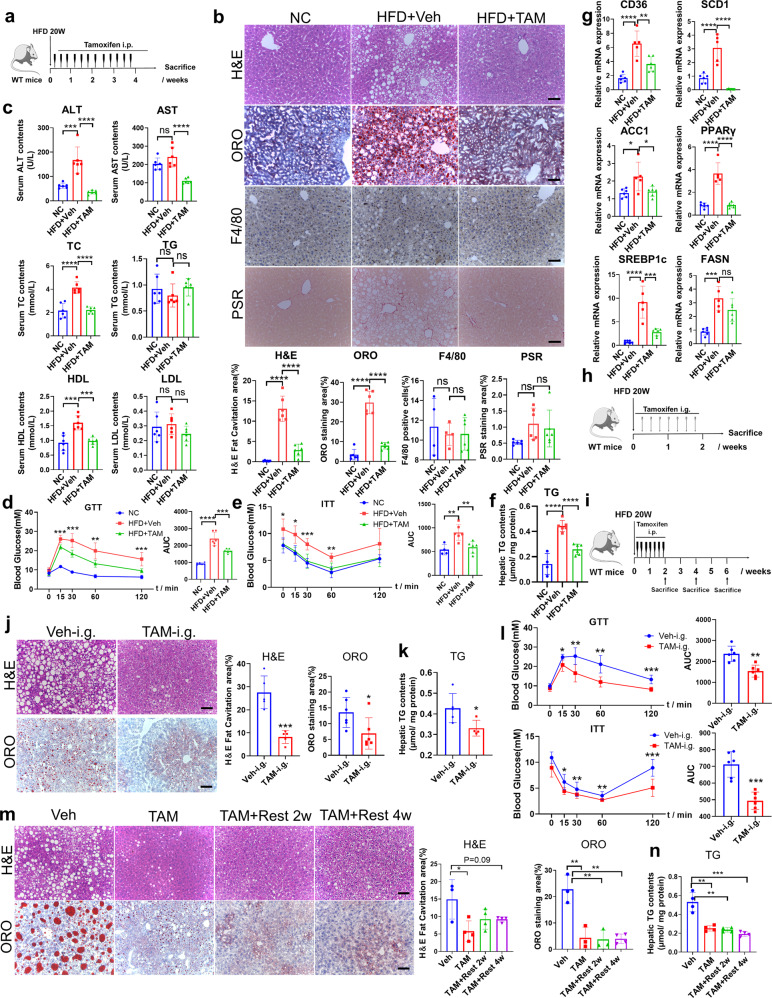
Tamoxifen ameliorated NAFLD in HFD-induced model. **a** Dosing scheme of tamoxifen on male C57BL/6 mice fed with high-fat diets for 20 weeks. Dose of tamoxifen: 100 mg/kg. **b** Liver sections from tamoxifen group and vehicle group in mice fed with high fat diets were performed H&E, ORO, F4/80 IF, and PSR staining. Mice fed with normal diets and administrated with vehicle were as controls. H&E fat cavitation area, ORO staining area, F4/80 positive cells percentage and PSR staining area were quantitatively compared. Scale bar:100 μm. **c** Serum analysis of ALT, AST, TC, TG, and LDL in high fat diets-induced mice administrated with tamoxifen or vehicle. Mice fed with normal diets and administrated with vehicle were as controls. **d** GTT experiment was performed on control mice or high fat diets-induced mice administrated with tamoxifen or vehicle and area under curve was quantitatively compared. **e** ITT experiment was performed on control mice or high fat diets-induced mice administrated with tamoxifen or vehicle and area under curve was quantitatively compared. **f** Hepatic TG levels were examined and normalized by protein levels. **g** RNA was extracted from isolated hepatocytes of control mice or high fat diets-induced mice administrated with tamoxifen or vehicle and expression of lipogenic genes was determined by RT-qPCR with β-actin as an internal control. **h** Dosing scheme of oral tamoxifen administration on male C57BL/6 mice fed with high-fat diets for 20 weeks. Dose of tamoxifen: 100 mg/kg. **i** Male C57BL/6 mice fed with high-fat diets for 20 weeks were administrated with 100 mg/kg tamoxifen intraperitoneally for 2 weeks and ceased treatment for 2 or 4 weeks. **j** Liver sections from male HFD-induced mice administrated with tamoxifen or vehicle orally for 2 weeks were performed H&E and ORO staining and H&E fat cavitation area, and ORO staining area was quantitatively compared. Scale bar:100 μm. **k** Hepatic TG levels were examined and normalized by protein levels. **l** GTT and ITT experiments were performed on male HFD-induced mice administrated with tamoxifen or vehicle orally for 2 weeks and area under curve was quantitatively compared. **m** Liver sections from male HFD-induced mice administrated with tamoxifen or vehicle for 2 weeks and ceased tamoxifen treatment for 2 or 4 weeks were performed H&E and ORO staining and H&E fat cavitation area and ORO staining area was quantitatively compared. Scale bar: 100 μm. **n** Hepatic TG levels were examined and normalized by protein levels. Bars = means ± SD; *n* = 3 to 6; ns, no significance; *P < 0.05; **P < 0.01; ***P < 0.001; ****P < 0.0001

In order to establish NAFLD models with metabolic abnormality, male mice were fed with HFD diet for 20 weeks. Then, 100 mg/kg tamoxifen was administrated intraperitoneally three times a week for 4 weeks (Fig. 4a). Compared to the control, mice fed with HFD diet showed increased body weight and liver weight, which were reduced by tamoxifen evidently (Supplementary Fig. S4a, b). Consistent with the findings in MCD and CDAA diet-induced models, tamoxifen alleviated hepatic steatosis but did not alter the phenotypes of inflammation and fibrosis in HFD-induced mice (Fig. 4b, f). Additionally, tamoxifen treatment decreased serum levels of ALT, AST, TC, HDL-C, and LDL-C instead of TG (Fig. 4c), suggesting that tamoxifen ameliorated lipid-induced liver injury and decreased serum cholesterol contents (Fig. 4c). Moreover, GTT and ITT assays confirmed that mice fed with HFD diet recovered significantly from glucose and insulin intolerance following tamoxifen treatment (Fig. 4d, e). Afterwards, we extracted RNA from isolated primary hepatocytes of fatty liver. RT-qPCR analyses demonstrated that HFD-triggered upregulation of lipogenic genes, including Srebp1c, CD36, SCD1, FASN, ACC1, and PPARγ, was neutralized by using tamoxifen (Fig. 4g). To reconfirm the molecular changes in tamoxifen-treated liver, CD36, SCD1, CCL2, CXCL10, Col3a1, and Timp1 were measured by RT-qPCR in total liver tissue and all of them were downregulated by tamoxifen (Supplementary Fig. S4c).

### Oral administration of tamoxifen effectively improves HFD-induced NAFLD

Considering that tamoxifen is usually delivered orally in clinical practice, we investigated whether oral tamoxifen administration alleviated NAFLD in HFD-induced NAFLD mice. NAFLD mice were administrated with 100 mg/kg tamoxifen or vehicle by oral gavage every other day for 2 weeks (Fig. 4h). Oral tamoxifen administration didn’t change body weight and liver weight (Supplementary Fig. S5a). However, serum ALT, TC, HDL, and LDL levels were decreased and glucose and insulin intolerance were improved notably (Fig. 4l and Supplementary Fig. S5b). Consistent with previous results, oral tamoxifen administration alleviated hepatic steatosis (Fig. 4j, k). These results collectively suggested that either intraperitoneal injection or oral administration of tamoxifen can effectively improve hepatic steatosis and metabolic dysfunction.

### The therapeutic effect of tamoxifen lasts after cessation of treatment

Whether the therapeutic effect of tamoxifen can be sustained after drug withdrawal remains unknown. To address this issue, we administrated 100 mg/kg tamoxifen intraperitoneally every other day for 2 weeks on HFD-induced NAFLD mice. Mice were sacrificed 2 or 4 weeks after the treatment (Fig. 4i). The body weight decreased in a time-dependent manner but liver weight showed no difference among different groups (Supplementary Fig. S6a, b). Intriguingly, serum ALT and AST levels decreased after drug withdrawal however serum TC, TG, HDL, and LDL levels increased (Supplementary Fig. S6c). Likewise, after drug withdrawal, the glucose intolerance was restored gradually and insulin tolerance remained unchanged (Supplementary Fig. S6d). However, drug withdrawal did not increase hepatic steatosis (Fig. 4m, n). The above results indicated that the therapeutic effect of tamoxifen on hepatic steatosis and liver injury could last at least 4 weeks.

### Short-term tamoxifen treatment alleviates HFD-induced hepatic steatosis

To verify whether short-term tamoxifen treatment is also effective, pair-fed male mice were administrated with 100 mg/kg tamoxifen intraperitoneally every other day for 14 days following 20-week HFD feeding (Supplementary Fig. S7a). After tamoxifen administration, the body weight was lost (Supplementary Fig. S7b, c) and the glucose and insulin intolerance were ameliorated in short-term HFD-treated mice (Supplementary Fig. S7d, e). Consistently, hepatic steatosis was alleviated (Supplementary Fig. S7f, h) and serum levels of ALT, AST, TC, HDL-C, and LDL-C were lowered by tamoxifen treatment as well (Supplementary Fig. S7g). To verify the involved mechanism, we performed RT-qPCR and found that tamoxifen didn’t increase mRNA expression of TG export and glucogenic-related genes (Supplementary Fig. S7i). These results collectively demonstrated that short-term tamoxifen administration showed the same effect on the treatment of fatty liver disease.

### The therapeutic effect of tamoxifen on metabolic dysfunction is dose-dependent

To evaluate whether the therapeutic effect of tamoxifen is dose-dependent, we analyzed mice administrated with different doses of tamoxifen (10 mg/kg, 50 mg/kg, and 100 mg/kg) following 20-week HFD feeding. Notably, administration of tamoxifen at different doses did not change liver weight or body weight (Supplementary Fig. S8a) but reduced serum levels of ALT, AST, TC, and HDL-C (Supplementary Fig. S8b) and alleviated hepatic steatosis without difference (Supplementary Fig. S8c–e). To further investigate the therapeutic effect of tamoxifen at lower doses, we analyzed mice administrated with tamoxifen for 1 or 5 mg/kg following 20-week HFD feeding. Lower-dose tamoxifen treatment didn’t change body weight and liver weight (Supplementary Fig. S9a) and did not alter serum ALT, AST, TC, TG, and HDL concentrations and glucose and insulin tolerance (Supplementary Fig. S9b, c). H&E and ORO staining and hepatic TG measurement revealed that lower-dose tamoxifen decreased hepatic steatosis and hepatic TG and 5 mg/kg tamoxifen treatment decreased more TG deposition than that of 1 mg/kg tamoxifen treatment (Supplementary Fig. S9d, e), revealing that tamoxifen treatment could alleviate hepatic steatosis in a dose-dependent manner. The above results suggested that the therapeutic effect of tamoxifen on metabolic dysfunction was dose-dependent and the dose range is lower for steatosis for tamoxifen than for other metabolic parameters.

### Tamoxifen has no sex disparity in fatty liver treatment

**Fig. 5:**
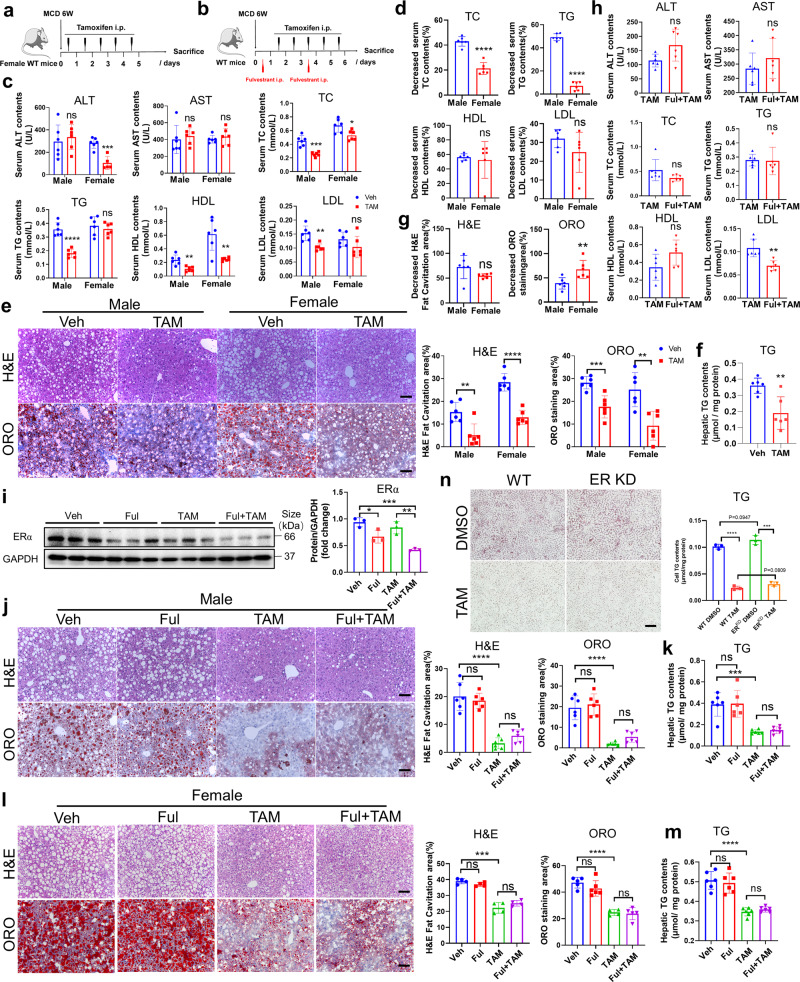
The therapeutic role of tamoxifen was independent of sex disparity and estrogen receptor. **a** Dosing scheme of tamoxifen on female C57BL/6 mice fed with MCD diets for 6 weeks. Dose of tamoxifen: 100 mg/kg. **b** Dosing scheme of tamoxifen combined with ER antagonist-fulvestrant on C57BL/6 mice fed with MCD diets for 6 weeks. Dose of tamoxifen: 100 mg/kg. **c** Serum analysis of ALT, AST, TC, TG, HDL, and LDL in MCD diets-induced male and female mice administrated with tamoxifen or vehicle. **d** Comparison of the decreased serum TC, TG, HDL, and LDL concentrations caused by tamoxifen between male and female mice. **e** Liver sections from male or female MCD diets-induced mice administrated with tamoxifen or vehicle were performed H&E and ORO staining. H&E fat cavitation area and ORO staining area were quantitatively compared. Scale bar: 100 μm. **f** Hepatic TG measurement in female MCD-induced NASH mice administrated with tamoxifen or vehicle. **g** Comparison of the decreased H&E fat cavitation area and ORO staining area caused by tamoxifen between male and female mice. **h** Serum analysis of ALT, AST, TC, TG, HDL, and LDL in male MCD diets-induced mice administrated with tamoxifen or tamoxifen + fulvestrant. **i** Protein levels of estrogen receptor in liver tissues from NASH mice administrated with tamoxifen w/o fulvestrant were determined by western blotting and quantitatively compared. **j** Liver sections from male MCD diets-induced mice administrated with vehicle, fulvestrant, tamoxifen or tamoxifen + fulvestrant were performed H&E and ORO staining. H&E fat cavitation area and ORO staining area were quantitatively compared. Scale bar: 100 μm. **k** Hepatic TG levels were examined on male MCD diets-induced mice administrated with vehicle, fulvestrant, tamoxifen, or tamoxifen + fulvestrant. **l** Liver sections from female MCD diets-induced mice administrated with vehicle, fulvestrant, tamoxifen or tamoxifen + fulvestrant were performed H&E and ORO staining. H&E fat cavitation area and ORO staining area were quantitatively compared. Scale bar: 100 μm. **m** Hepatic TG levels were examined on female MCD diets-induced mice administrated with vehicle, fulvestrant, tamoxifen, or tamoxifen + fulvestrant. **n** WT and ER knockdown AML12 cells were treated with PA for 36 h and then treated with 40 μM tamoxifen for another 36 h. ORO staining and cellular TG measurement were performed. Scale bar:100 μm. Bars = means ± SD; *n* = 3–6; ns, no significance; *P < 0.05; **P < 0.01; ***P < 0.001; ****P < 0.0001

As mentioned previously, tamoxifen is a SERM. RNA-sequence of tamoxifen-treated hepatocytes confirmed the inactivation of estrogen-related signaling pathways (Supplementary Fig. S10a). To clarify whether the therapeutic effect of tamoxifen was gender or ER-dependent, we first discovered the influence of sex disparity in tamoxifen treatment. Both male and female mice were administrated with 100 mg/kg tamoxifen as previous description (Figs. 3a and 5a). Except for body weight, no significant change of liver weight and liver to body weight ratio was inspected between male and female mice following tamoxifen treatment (Supplementary Fig. S10b). Tamoxifen treatment reduced serum levels of TC, TG, HDL-C, and LDL-C in male mice and decreased ALT, TC, and HDL-C in female mice (Fig. 5c). Then, we compared the decreased ratio of serum levels of TC, TG, HDL-C and LDL-C between male and female mice. Notably, TC and TG decreased less in tamoxifen-treated female mice than in male ones while the decrease in serum HDL-C and LDL-C showed no difference (Fig. 5d). H&E and ORO staining and hepatic TG measurement revealed that tamoxifen attenuated hepatic steatosis in both male and female mice (Figs. 3h and 5e, f). Moreover, the reduction of fat cavitation showed no difference between male and female mice, however, the reduction of ORO staining areas in female mice is greater than it in male ones (Fig. 5g), which was inconsistent with the findings shown in Fig. 5d, implying sex disparity may probably have no influence on tamoxifen treatment.

### Tamoxifen treats hepatic steatosis estrogen receptor independently

To clarify whether tamoxifen ameliorates fatty liver through ER, we employed a pharmacological ER antagonist (fulvestrant) to facilitate ER degradation.^28,29^ As shown in Fig. 5b, 100 mg/kg fulvestrant was administrated twice during 100 mg/kg tamoxifen treatment. WB results showed that fulvestrant could effectively reduce hepatic ER protein expression (Fig. 5i). Interestingly, fulvestrant did not affect ALT, AST, TC, TG, and HDL-C in tamoxifen-treated mice (Fig. 5h). Additionally, fulvestrant made no change to the therapeutic effect of tamoxifen on hepatic steatosis (Fig. 5j, k). In female mice, fulvestrant didn’t change body weight or liver weight (Supplementary Fig. S10c). Fulvestrant decreased serum TC and HDL levels but didn’t change serum levels of ALT, AST, TG and LDL (Supplementary Fig. S10f). Similarly, fulvestrant administration exerted no influence on the therapeutic effect of tamoxifen on hepatic steatosis in female mice (Fig. 5l, m). To exclude the potential interference of fulvestrant on extrahepatic tissues, we use ER shRNA to suppress the expression of ER on AML12 cell line. RT-qPCR and WB results suggested that ER shRNA decreased cellular ER expression notably (Supplementary Fig. S10d, e). Consistently, the effect of tamoxifen on decreasing TG accumulation was not affected by ER knockdown (Fig. 5n). Above results collectively proved that the treatment of tamoxifen on lipid metabolism is independent of ER.

### Tamoxifen impedes hepatic steatosis by inhibiting JNK/MAPK pathway

**Fig. 6:**
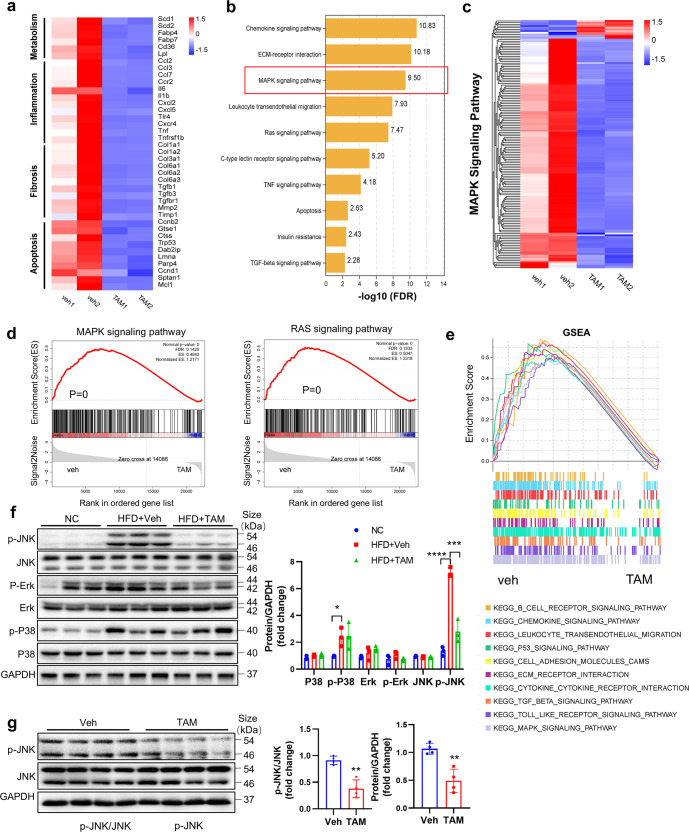
Tamoxifen inhibited JNK/MAPK signaling pathway. RNA was extracted from isolated hepatocytes of male MCD diets-induced NASH mice administrated with tamoxifen or vehicle and RNA-seq was performed. Fold change > 1.5, *P* < 0.05 or FDR < 0.25 were considered significant. **a** Gene expression heatmap of lipid metabolism, inflammation, fibrosis and apoptosis-related genes. **b** KEGG analysis of DEGs. **c** Heatmap of MAPK signaling pathway-related DEGs. **d** GSEA analysis of MAPK and Ras signaling pathways. **e** GSEA analysis of MAPK signaling pathway and inflammation, fibrosis and apoptosis-related signaling pathways. Fig. **a**–**e** were generated on Omicsmart platform (https://www.omicsmart.com/). **f** Protein levels of three key MAPK molecules-P38, JNK, and ERK were determined by western blotting and quantitatively compared. **g** Total protein was extracted from liver tissues in normal diets-fed mice administrated with tamoxifen or vehicle for 8 weeks. Protein levels of JNK and p-JNK were determined by western blotting and quantitatively compared. Bars = means ± SD; *n* = 3 to 4; *P < 0.05; **P < 0.01; ***P < 0.001; ****P < 0.0001

In order to elucidate the mechanisms involved in the treatment of tamoxifen on NAFLD, RNA-sequence was performed in isolated hepatocytes of fatty liver. Correlation heatmap and principal component analysis showed clearly separated clusters for the multiple samples (Supplementary Fig. S11a, b). Volcano plot suggested that most differentially expressed genes (DEGs) were downregulated after tamoxifen treatment (Supplementary Fig. S11c). Consistent with RT-qPCR results, gene heatmap revealed that genes associated with lipid metabolism, inflammation, fibrosis and apoptosis were inactivated by tamoxifen (Fig. 6a). In KEGG analysis of DEGs, we found that inflammation, fibrosis, apoptosis and insulin resistance pathways were enriched (Fig. 6b). More importantly, MAPK signaling pathway and its key upstream regulator-Ras signaling, were obviously enriched as well (Fig. 6b). The heatmap further confirmed that most of the genes in MAPK signaling pathway were downregulated by tamoxifen treatment (Fig. 6c). Besides, GSEA analysis showed that MAPK and Ras signaling pathway (Fig. 6d), coupled with other signaling pathways such as chemokine signaling pathway, toll like receptor signaling pathway, TGF-β signaling pathway and P53 signaling pathway were extensively inhibited by tamoxifen treatment (Fig. 6e). Interestingly, GSEA analysis proved that tamoxifen facilitated fatty acid degradation instead of inhibiting fatty acid biosynthesis (Supplementary Fig. S11d). In addition, inflammation and fibrosis-related signaling pathways were found to be downregulated notably in tamoxifen-treated hepatocytes (Supplementary Fig. S11e, f). Thereafter, western blot analyses confirmed the activation of JNK and P38 in hepatocytes of fatty liver and only JNK pathway was inhibited by tamoxifen treatment (Fig. 6f). However, ERK phosphorylation was not affected by tamoxifen administration (Fig. 6f). Besides, we found that tamoxifen also decreased hepatic JNK phosphorylation in mice fed with 8-week normal diet (Fig. 6g). Overall, these data identified the JNK/MAPK pathway as the candidate signaling pathway regulated by tamoxifen in NAFLD pathogenesis.

### Activation of JNK/MAPK pathway abolishes the effect of tamoxifen on hepatic steatosis treatment

**Fig. 7:**
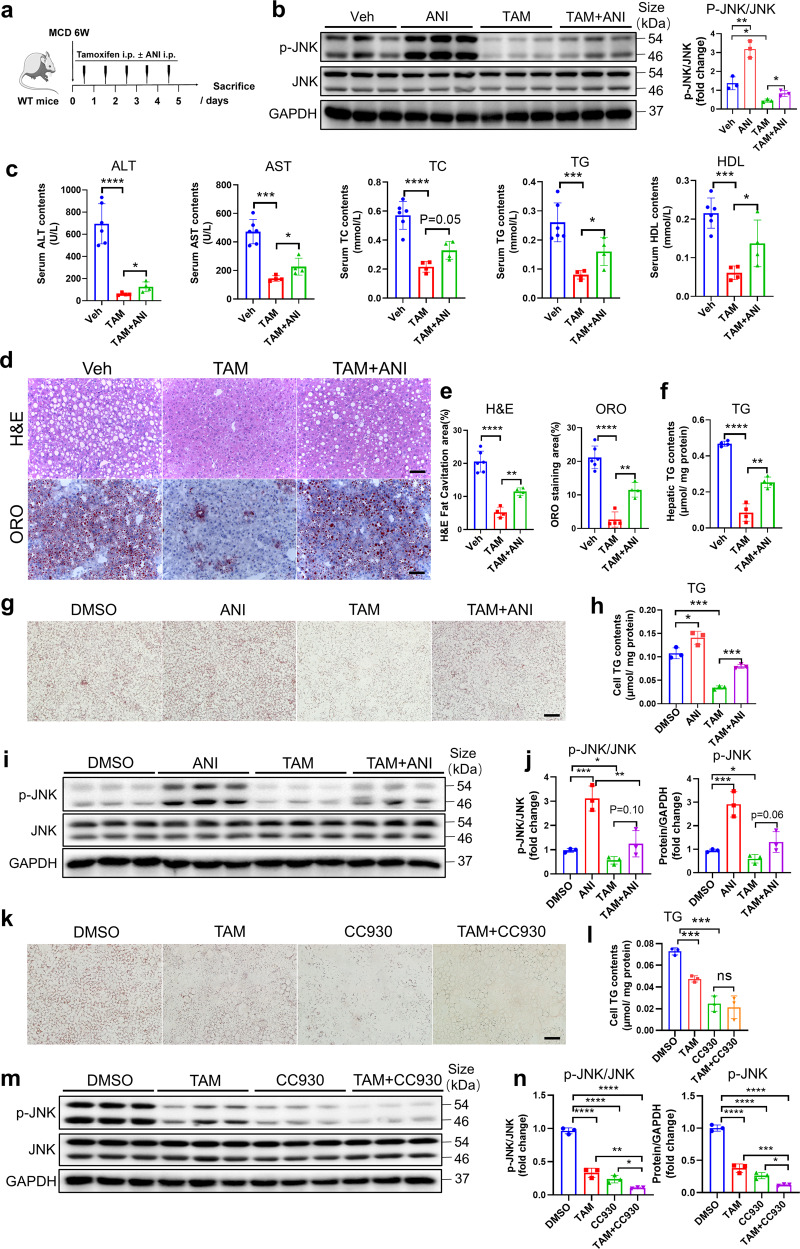
Pharmacological activation of JNK/MAPK signaling pathway partly abolished the therapeutic effect of tamoxifen. **a** Dosing scheme of tamoxifen coupled with JNK activator-anisomycin (ANI) on male C57BL/6 mice fed with MCD diets for 6 weeks. Dose of tamoxifen: 100 mg/kg. Dose of anisomycin: 50 mg/kg. **b** Total protein was extracted from liver tissues in mice administrated with vehicle, ANI, tamoxifen or tamoxifen + ANI. Protein levels of JNK and p-JNK were determined by western blotting and quantitatively compared. **c** Serum analysis of ALT, AST, TC, TG, and HDL in mice administrated with vehicle, tamoxifen or tamoxifen + ANI. **d** Liver sections from MCD diets-induced mice administrated with vehicle, tamoxifen, or tamoxifen + ANI were performed H&E and ORO staining. Scale bar:100 μm. **e** H&E fat cavitation area and ORO staining area were quantitatively compared. **f** Hepatic TG levels were measured and normalized by protein levels. **g** AML12 cells were stimulated by 0.3 mM PA and treated with 40 μM tamoxifen. 10 μM anisomycin or DMSO was added 2 h before tamoxifen treatment. ORO staining was performed. Scale bar: 100 μm. **h** Cellular TG contents were measured and quantitatively compared. **i** Total proteins were extracted from AML12 cells treated with DMSO/ ANI/ tamoxifen/ tamoxifen+ANI. Protein levels of JNK and p-JNK were determined by western blotting. **j** The ratio of p-JNK/JNK and p-JNK/GAPDH were quantitatively compared. **k** AML12 cells were stimulated by 0.3 mM PA and treated with 40 μM tamoxifen. 30 μM CC930 or DMSO were added 2 h before tamoxifen treatment. ORO staining was performed. Scale bar: 100 μm. **l** Cellular TG contents were measured and quantitatively compared. **m** Total proteins were extracted from AML12 cells treated with DMSO/tamoxifen/CC930/tamoxifen+CC930. Protein levels of JNK and p-JNK were determined by western blotting. **n** The ratio of p-JNK/JNK and p-JNK/GAPDH were quantitatively compared. Bars = means ± SD; *n* = 3–6; ns, no significance; *P < 0.05; **P < 0.01; ***P < 0.001; ****P < 0.0001

To prove JNK/MAPK pathway is required for the treatment of tamoxifen on NAFLD, we applied a pharmacological JNK activator-anisomycin (ANI).^30^ Male mice fed with MCD diet were injected with 100 mg/kg tamoxifen and 50 mg/kg ANI for 5 consecutive days (Fig. 7a). Firstly, we proved that anisomycin administration could increase JNK phosphorylation in liver tissues (Fig. 7b). Activation of JNK by ANI restored the decreased body weight and liver weight caused by tamoxifen (Supplementary Fig. S12a). In addition, tamoxifen-triggered reductions of ALT, AST, TC, TG, and HDL-C were all reversed by ANI administration (Fig. 7c). Consistently, ANI partially abolished the therapeutic effect of tamoxifen on hepatic steatosis (Fig. 7d–f). We further proved the effect of ANI in AML12 cell line. ORO staining and TG measurement indicated that ANI increased TG accumulation in the absence or presence of tamoxifen (Fig. 7g, h) by increasing JNK phosphorylation (Fig. 7i, j), suggesting that JNK activation aggravated hepatic steatosis and abrogated the therapeutic effect of tamoxifen in vitro. RT-qPCR analyses suggested that ANI treatment increased the expression of lipogenic genes (Srebp1c, FASN, ACC1), which were downregulated by tamoxifen (Supplementary Fig. S12b). In contrast, CC930, a JNK inhibitor, could effectively decrease JNK phosphorylation and alleviate hepatocyte steatosis (Fig. 7k–n). Taken together, these results confirmed tamoxifen-treated hepatic steatosis by inhibiting JNK/MAPK signaling.

## Discussion

Tamoxifen, a selective estrogen receptor modulator, was approved by the Food and Drug Administration for the treatment of breast cancer since 1977.^31^ Despite its success in cancer chemotherapy, the multiple roles of tamoxifen in regulating estrogen receptors should be noticed. In cardiovascular system, tamoxifen acts as an estrogen agonist to lower the incidence of fatal myocardial infarction, but on the other hand, tamoxifen also shows antiestrogenic effects on triggering coronary artery constriction.^32^ In liver, tamoxifen exhibits hepatoprotective roles as an estrogen agonist in acute and chronic liver injuries,^33,34^ while impedes cholangiocyte proliferation and accelerates their apoptosis as an estrogen antagonist. This paradoxical phenomenon may be attributed to cholangiocytes expressing both ERα and ERβ subtypes while hepatocytes only express ERα.^35^ In recent years, tamoxifen non-estrogen receptor-mediated molecular targets have been gradually paid much more attention. Tamoxifen inhibits PKC activity to restrict tumor growth and promote cancer cell apoptosis.^36^ A few PKC isoforms have been proven to be activated in NASH, so tamoxifen may also alleviate NASH through PKC inhibition and PKC is also a potent upstream regulator of MAPK pathway.^37^ In addition, tamoxifen was proven to exert its effectiveness through some other targets such as androgen receptor,^38^ estrogen-related receptor gamma^39^, and mitogen-activated protein kinase 8.^36^ These findings implicate that the pharmacological effects of tamoxifen are so complicated depending on tissue specificity and dose effect and its therapeutic roles in the treatment of different diseases being currently investigated preclinically.^40,41^

Occasionally, during the induction of CreER recombinase activity by tamoxifen, we found that tamoxifen diminished serum lipid contents and inhibited hepatic lipid accumulation simultaneously in mice. This phenomenon arouses our great interest. We conjectured if tamoxifen could treat NAFLD or NASH in mice. To confirm our hypothesis, we first evaluated the effect of tamoxifen on lipotoxicity. As expected, tamoxifen protected hepatocytes against sodium palmitate-induced lipotoxicity in all three cell lines and primary hepatocytes without increasing hepatotoxicity. Afterwards, we administrated tamoxifen continuously for 8 weeks in male and female mice fed with normal diets. We observed the role of tamoxifen in decelerating lipid accumulation in blood and in liver of normal mice and found that tamoxifen decreased serum cholesterol contents and inhibited hepatic lipid accumulation. Importantly, continuous tamoxifen administration did not worsen liver function at all. To testify the therapeutic effect of tamoxifen on NAFLD or NASH, we established MCD, CDAA, and HFD diets-induced metabolic disordered models. Consistently, tamoxifen decreased hepatic TG and serum TC contents and the mRNA expressions of lipogenesis, inflammation, and fibrosis-related genes in all three models. In addition, tamoxifen alleviated insulin intolerance in HFD-induced models, implicating that tamoxifen effectively ameliorated NAFLD. Besides, we examined extrahepatic adipose tissues and found that tamoxifen decreased adipocyte area but did not alter insulin secretion (Supplementary Fig. S13a, b). This finding indicated that tamoxifen may alleviate NAFLD through enhancing lipolysis and increasing insulin sensitivity instead of insulin secretion.

In view that tamoxifen is an oral medicine in clinics, we proved that oral tamoxifen had the same therapeutic effect compared to intraperitoneal injection, suggesting that different routes of tamoxifen administration would not affect its therapeutic effect. Although we proved that tamoxifen ameliorated NAFLD obviously, whether the therapeutic effect could sustain after treatment cessation remained controversial. After drug withdrawal, the therapeutic effect of tamoxifen on hepatic steatosis was sustained for 4 weeks, however, the influence on other indicators such as serum TC, TG, HDL, and LDL levels and glucose and insulin tolerance gradually disappeared. These findings give us hints that intermittent tamoxifen administration can be adopted to reach lasted and satisfied effects in NAFLD treatment.

Tamoxifen was proven to be an appetite suppressor in preclinical animal models and in clinical patients.^42^ To avoid this undesired effect, mice administrated with tamoxifen were strictly pair-fed. In view that loss of body weight contributes to the improvement of NAFLD,^43^ the changes in body weight, liver weight, and liver-to-body weight ratio were consistently monitored while tamoxifen was administrated. In normal, MCD and CDAA-treated mice, tamoxifen improved fatty liver without affecting body weight, proving weight loss was not the reason for the decreased lipid accumulation in tamoxifen-treated mice. It is worth noting that, tamoxifen is a canonical ER modulator. However, no difference was found in mice of different genders following tamoxifen treatment. Besides, blocking ER didn’t alter the therapeutic effect of tamoxifen as well, proving tamoxifen improved NAFLD through non-estrogen targets. We previously reported the importance of TAK1-mediated MAPK signaling pathway in regulating NASH.^44^ In this study, we confirmed that JNK/MAPK was required for tamoxifen to treat fatty liver disease, as tamoxifen inactivated JNK/MAPK signaling and JNK activator abolished the therapeutic effect of tamoxifen. As for how tamoxifen affects JNK signaling, in vitro experiments revealed that the phosphorylation of canonical upstream regulators, TAK1 and ASK1, was inhibited by tamoxifen (data not shown). Combined our results with published manuscripts, we speculate that tamoxifen may suppress JNK activation through TAK1 or ASK1 dephosphorylation which needs to be further determined. Intriguingly, tamoxifen decreased expressions of lipid uptake, de novo lipogenesis, fatty acid oxidation, and lipid export-related genes simultaneously, suggesting that tamoxifen could suppress hepatic lipid metabolism comprehensively but whether tamoxifen alleviated NAFLD through lipid metabolism gene expression modulation directly needs further investigation.

Intriguingly, our findings seem inconsistent with a few preclinical studies. In the 1990s, long-term tamoxifen treatment was found to induce NAFLD/NASH in female patients with breast cancer.^10,12^ In recent years, a few publications revealed that low-dose tamoxifen administration for 5 consecutive days increased hepatic lipid accumulation by enhancing fatty acid synthesis.^15^ Conversely, some findings demonstrated that tamoxifen protected hepatocytes against lipotoxicity and steatosis.^20,21^ Lelliott et al. also reported that tamoxifen inhibited fatty acid synthesis in the presence of hepatic steatosis.^45^ It seems that the impact of tamoxifen on lipid metabolism is still elusive. In addition, Ceasrine et al. reported that tamoxifen had significant and sustained effects on glucose tolerance which was in line with our in vivo results.^46^ The reason why our results are contrary to some of the findings mentioned above, is probably that we administrated tamoxifen in a short term (1–4 weeks) and at a higher dose (100 mg/kg). However, whether high-dose tamoxifen administration is safe and its potential side effects need to be determined. In our study, at least serum ALT and AST analyses showed that short-term high-dose tamoxifen treatment did not cause hepatoxicity in mice. In order to evaluate the safety and side effects of high-dose tamoxifen administration in clinics, we tried to translate mice dose to human dose through normalization to body surface area (BSA).^47^ After calculation, the human equivalent dose (HED) of tamoxifen usage we estimated is 8 mg/kg (300 mg/m^2^). Skapek et al. performed a phase II study on children with desmoid fibromatosis and proved that tamoxifen administration at a maximum dose of 300 mg per day caused few serious side effects.^48^ In addition, high-dose tamoxifen is also used to treat malignant gliomas (240 mg/d)^49^ and cryptococcal meningitis (300 mg/d).^50^ Trump et al. conducted a phase I clinical trial to investigate the regulatory role of high-dose, oral tamoxifen in P-glycoprotein-mediated drug resistance and found that tamoxifen given at a dose of 300 mg/m^2^ per day for 12 days following a loading dose of 400 mg/m^2^ did not cause dose-limiting toxicity.^51^ In other studies, breast cancer patients with renal cell carcinoma were well tolerated with tamoxifen administrated at a dose of 200 mg/m^2^ /day for up to 1 year.^52,53^ Even if considering the safety of the use of high-dose tamoxifen, we proved that low-dose tamoxifen (i.e., 1, 5 mg/kg), which was probably insufficient to restore the glucose intolerance and liver malfunction, was still effective in treating hepatic steatosis in NAFLD mice. Therefore, short-term tamoxifen administration seems to be realistic to treat NAFLD in clinics.

## Materials and methods

### Cell culture

Cell lines were obtained from the Cell Bank of Chinese Academy of Sciences (Shanghai, China). Huh7 and HepG2 cell lines were grown in RPMI1640 or high-glucose DMEM medium (Gibco, USA) supplemented with 10% fetal bovine serum (FBS) (Gibco, USA) and 100 U/ml penicillin and 0.1 mg/ml streptomycin (MI00614, Mishushengwu, Xi’an, China). AML12 cell line was grown in DMEM: F12 medium (11330, Invitrogen, USA) supplemented with 10% FBS, 1% ITS liquid media supplement (I3146, Sigma, USA), and 40 ng/ml dexamethasone. All cell lines were kept in a humidified incubator at 37 °C and 5% CO_2_. Cells were used from third to tenth passage in each experiment. Primary hepatocytes were isolated from 8-week-old male C57BL/6 mice and were cultured in HM medium (ScienCell, USA) and seeded in six-well plates at 1 × 10^6^/well. To induce cellular lipotoxicity, 0.3 mM sodium palmitate (SYSJ-KJ, Kunchuang Biotechnology, Xi’an, China) was added into medium and vehicle was added as a control. After 36 h, tamoxifen dissolved in DMSO was added at a dose of 10, 20, 40 μM and kept for 36 h and DMSO was added as a control. To induce JNK activation in vitro, anisomycin (10 μM) was added 2 h before tamoxifen treatment. To inhibit JNK phosphorylation in vitro, 30 μM Tanzisertib (cc-930) (S8490, Selleck, China) was added 2 h before tamoxifen treatment. For cellular Oil Red O (ORO) staining, cells were cultured in six-well plates and after sodium palmitate and tamoxifen treatment, culture medium was removed, and cells were fixed with 4% paraformaldehyde for 30 min and washed with PBS three times. Then the cells were treated with 60% isopropanol for 5 min. Remove isopropanol, stain cells with ORO working solution (Servicebio Technology, Wuhan, China) for 10 min, and washed cells with PBS. Then the cells were stained with hematoxylin for 3–5 min and washed with PBS at least three times. Then we observed and took photos using an inverted phase contrast microscope (Olympus, X71, Japan). For cellular TC and TG tests, we purchased commercial kits from Pulilai Gene Technology Co., Ltd (Beijing, China) and followed the manufacturer’s instructions.

### Lentivirus transfection

Lentiviral particles bearing ER shRNA (gcTTTCTTTAAGAGAAGCATT) were purchased from GeneChem (Shanghai, China). AML12 cells were seeded in six-well plate at the concentration of 3 × 10^4^/ml. After 12 h, cells were transfected with ER shRNA viruses or control shRNA viruses. When the transfection efficiency reached 70–80%, 10 μg/ml puromycin was added to kill uninfected cells. Then, very passage was selected with 5 μg/ml puromycin until transfection efficiency reached nearly 100%.

### Mice

Male and female C57BL/6 mice (8 weeks old) were purchased from Weitong Lihua Experimental Animal Technology Co., Ltd (Beijing, China) and kept in specific pathogen-free animal house. The room temperature was controlled at around 23 °C and humidity was kept at 50–60% with a 12 h day/night cycle. The water was sufficient to obtain and in order to eliminate the potential appetite suppression role of tamoxifen, the mice with tamoxifen administration and their control mice were pair-fed unless specifically stated. In order to establish NASH mouse model, male or female mice were fed with a MCD diet (A02082002BR, Research Diets, New Brunswick, USA) for 6 weeks or a CDAA diet (A06071309, Research Diets, New Brunswick, USA) for 10 weeks. To establish a NAFLD mouse model, male or female mice were fed with a HFD (D12492, Research Diets, New Brunswick, USA) for 20 weeks. Tamoxifen was purchased from Sigma (T5648, USA), dissolved in corn oil, and stored at 4 °C for at most a week. Pharmacological ER antagonist fulvestrant (S1191) and JNK activator anisomycin (S7409) were purchased from Selleck (Shanghai, China). The dosing scheme was shown in figures and illustrated in the Results section.

All animal experiments were approved by the Animal Experiment Administration Committee of the Fourth Military Medical University (Xi’an, China) and proceeded under the instruction of Guide for the Care and Use of Laboratory Animals published by the National Institute of Health (publications 86-23, revised 1985).

### GTT and ITT

For GTT experiment, mice were fasted for 8 h and measured fasting blood glucose on tail vein using OneTouch Ultra glucometers (LifeScan). Then the mice were injected with 1 g/kg glucose intraperitoneally and blood glucose was recorded at 15, 30, 60, and 120 min. For ITT experiment, mice fasted for 6 h and measured fasting blood glucose. Then the mice were injected with 0.75 U/kg insulin subcutaneously and blood glucose was recorded at 15, 30, 60, 120 min.

### Histology

For H&E, PSR staining, and F4/80 IHC staining, fresh liver tissues or adipose tissues were fixed in 4% paraformaldehyde for at least 24 h and embedded with paraffin. Then the liver tissues were cut into 4–6 μm sections. H&E and PSR staining were conducted following the standard protocols. F4/80 IHC staining was performed as previously illustrated.^54^ For F4/80 IF and ORO staining, mice liver was fixed in 4% paraformaldehyde for 4 h and then transferred into 30% sucrose solution at 4 °C overnight. Then liver tissues were embedded with optimum cutting temperature compound (Sakura Finetek, Japan) and sectioned at 8–10 μm. For F4/80 IF, sections were embedded with primary antibody at 4 °C overnight, washed with PBS and embedded with fluorescent secondary antibody at room temperature, and counterstained with Hoechst 33258 (Sigma-Aldrich). For ORO staining, saturated Oil Red O solution was purchased from Servicebio technology and cryosections were stained following the manufacturer’s instructions.

### Serum biochemistry

Serum biochemical analysis was performed using commercial kits (Nanjing Jiancheng Biochemical, Nanjing, China) on an automatic biochemical analyzer (Chemray 240, Rayto, China) based on the instructions supplied by the manufacturer. Serum Insulin concentrations were examined using commercial ELISA kits (Jianglaibio, Shanghai, China).

### Cell isolation

Primary hepatocytes were isolated as previously described.^54^

### Cell viability

AML12 and Huh7 cell viability were examined using commercial kit (C0011, Beyotime, China).

### Gene expression profiling

Total RNA was extracted using Trizol Reagent from isolated primary hepatocytes of mice fed with MCD diets and administrated with tamoxifen or vehicle. Then RNA samples were sent to GENE DENOVO Biotechnology Co. (Guangzhou, China) for further sequencing. Briefly, eukaryotic mRNA was enriched by Oligo(dT) beads and fragmented into short fragments using fragmentation buffer and then reverse transcribed into cDNA. CDNA of about 200 bp was selected with AMPure XP beads, amplified through PCR, and purified with AMPure XP beads to construct cDNA library. RNA quality was assessed using an Agilent 2100 Bioanalyzer (Agilent Technologies, Palo Alto, CA, USA) and checked using RNase free agarose gel. The cDNA products were size selected by agarose gel electrophoresis, PCR amplified, and sequenced using Illumina Novaseq6000. Bioinformatic analysis was conducted on Omicsmart platform (https://www.omicsmart.com/). The raw sequences were deposited on the GEO website (GSE212148).

### RT-qPCR

We extracted total RNA from isolated hepatocytes or liver tissues using Trizol reagent (Invitrogen, USA) and followed the manufacturer’s protocols. Then the RNA was reverse-transcribed into cDNA using Evo M-MLV RT Premix for qPCR (Accurate Biology, Changsha, China). RT-qPCR was performed by using SYBR Green PCR Master Mix (Accurate Biology, Changsha, China) according to the manufacturer’s protocol. Relative gene expression levels were normalized to β-actin. The primers used in the current article were listed in Supplementary Table S1.

### Western blotting

Total protein was extracted with RIPA lysis buffer supplemented with 10 mM phenylmethanesulfonyl fluoride and quantified with BCA protein quantitative kit (Thermo Fisher Scientific, Rockford, IL). Protein samples were loaded and separated with SDS-PAGE gel electrophoresis and transferred onto polyvinylidene fluoride membranes. The membranes were blocked with 5% skimmed milk powder and incubated in primary antibodies at 4 °C overnight and washed with TBST. Then the membranes were incubated in secondary HRP-conjugated antibodies at room temperature for 2 h and washed with TBST. The protein signals were detected by ChemiDoc MP Imaging System (Bio-Rad, Hercules, CA, USA). The antibodies used in this article were listed in Supplementary Table S2.

### Statistics

Statistical analysis of data was performed using GraphPad Prism v8.3.0 and the results were expressed as the means ± SD. Data were tested for normality (Kolmogorov–Smirnov test) and homogeneity (Brown-Forsythe test) of variance. Difference between the two groups was analyzed by Student’s *t* test with normal distribution or by Mann-Whitney *U* test with skewed distribution. Difference among multiple groups was analyzed by one-way ANOVA with normal distribution followed by Tukey’s post hoc test or by Kruskal-Wallis test with skewed distribution. *P* values less than 0.05 were considered statistically significant. *p < 0.05; **p < 0.01; ***p < 0.001; ****p < 0.0001; ns, not significant.

## Supplementary information

suppl data

## Data Availability

All data supporting the findings of this study are available from the corresponding author upon reasonable request.
