# Supplementary material for: Short-term tamoxifen administration improves hepatic steatosis and glucose intolerance through JNK/MAPK in mice
Source: Signal Transduct Target Ther. 2023 Mar 3;8:94. doi: 10.1038/s41392-022-01299-y (PMC9981902; doi:10.1038/s41392-022-01299-y)
Supplement: Supplementary file 1 — suppl data [file 41392_2022_1299_MOESM1_ESM.docx]

Supplementary Materials for

Short-term tamoxifen administration improves hepatic steatosis and glucose intolerance through JNK/MAPK in mice

Zhiqiang Fang^1^, Hao Xu^1^, Juanli Duan, Bai Ruan, Jingjing Liu, Ping Song, Jian Ding, Chen Xu, Zhiwen Li, Kefeng Dou^*^, Lin Wang^*^

Correspondence to: [fierywang@163.com](mailto:fierywang@163.com); doukef@fmmu.edu.cn

**This PDF file includes:**

Figures S1 to S13

Tables S1 to S2

Figure. S1.


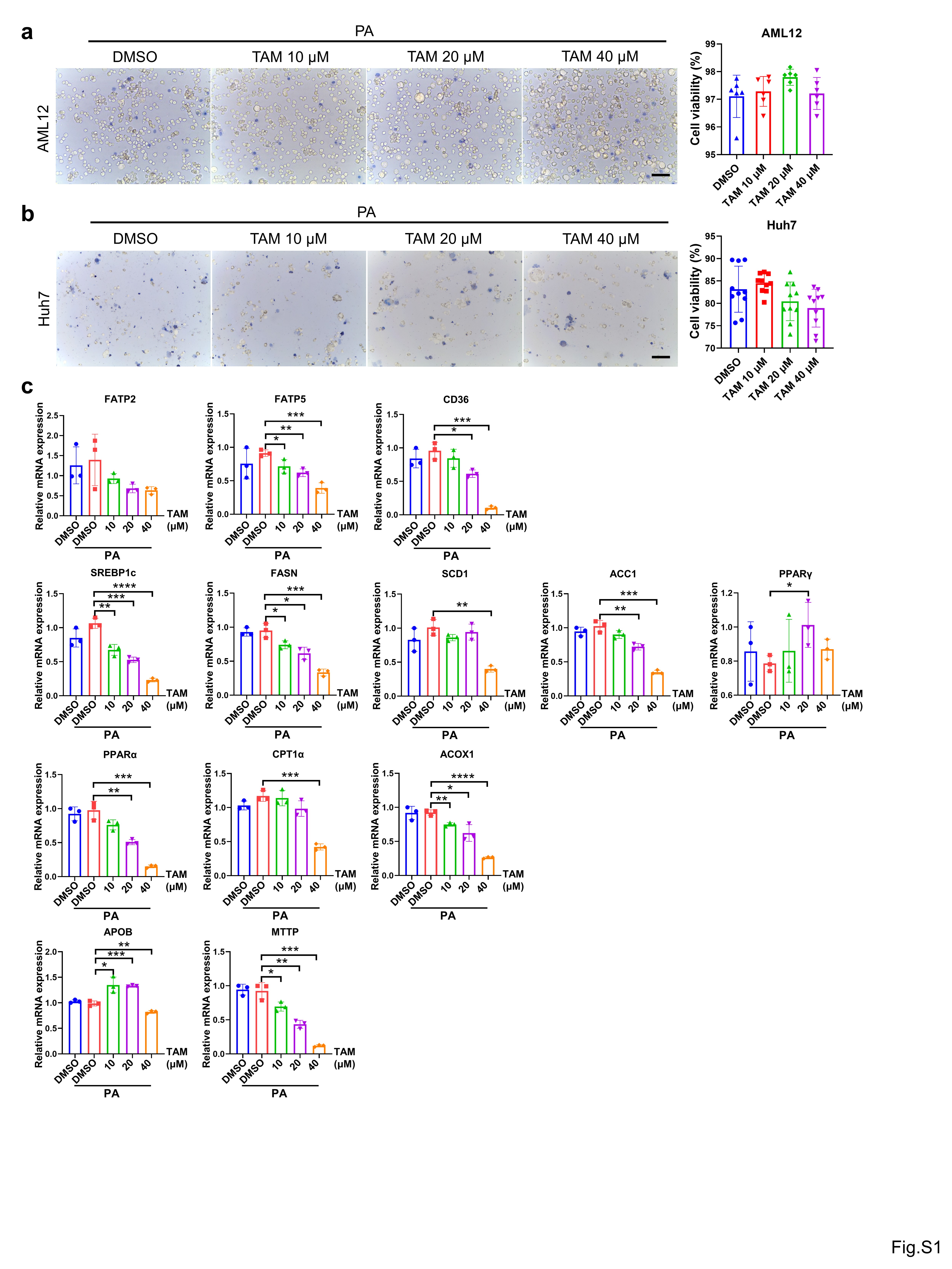


**Fig. S1** (**a**) AML12 cells were seeded in 6-well plates. After 12h, 0.3 mM sodium palmitate was added to the medium and after 36h, DMSO/tamoxifen (10, 20, 40 μM) was added. After 36h, cells were stained with trypan blue and pictured with light microscope. Cell viability represents the percentage of living cells. Scale bar: 100 μM. (**b**) Huh7 cells were seeded in 6-well plates. After 12h, 0.3 mM sodium palmitate was added to the medium and after 36h, DMSO/tamoxifen (10, 20, 40 μM) was added. After 36h, cells were stained with trypan blue and pictured with light microscope. Cell viability represents the percentage of living cells. (**c**) AML12 cells were seeded in 6-well plates. After 12h, 0.3 mM sodium palmitate was added to the medium and after 36h, DMSO/tamoxifen (10, 20, 40 μM) was added. After 36h, cells were harveated and total RNA was extracted and expressions of fatty acid uptake (FATP2, FATP5 and CD36), de novo lipogenesis (Srebp1c, FASN, SCD1, ACC1 and PPARγ), fatty acid oxidation (PPARα, CPT1α and ACOX1) and TG export (ApoB, MTTP)-related genes were determined by RT-qPCR with β-actin as an internal control. Scale bar: 100 μM. Bars = means ± SD. n=3 to 10. *P < 0.05; **P < 0.01; ***P <0.001; ****P<0.0001.

Figure. S2.


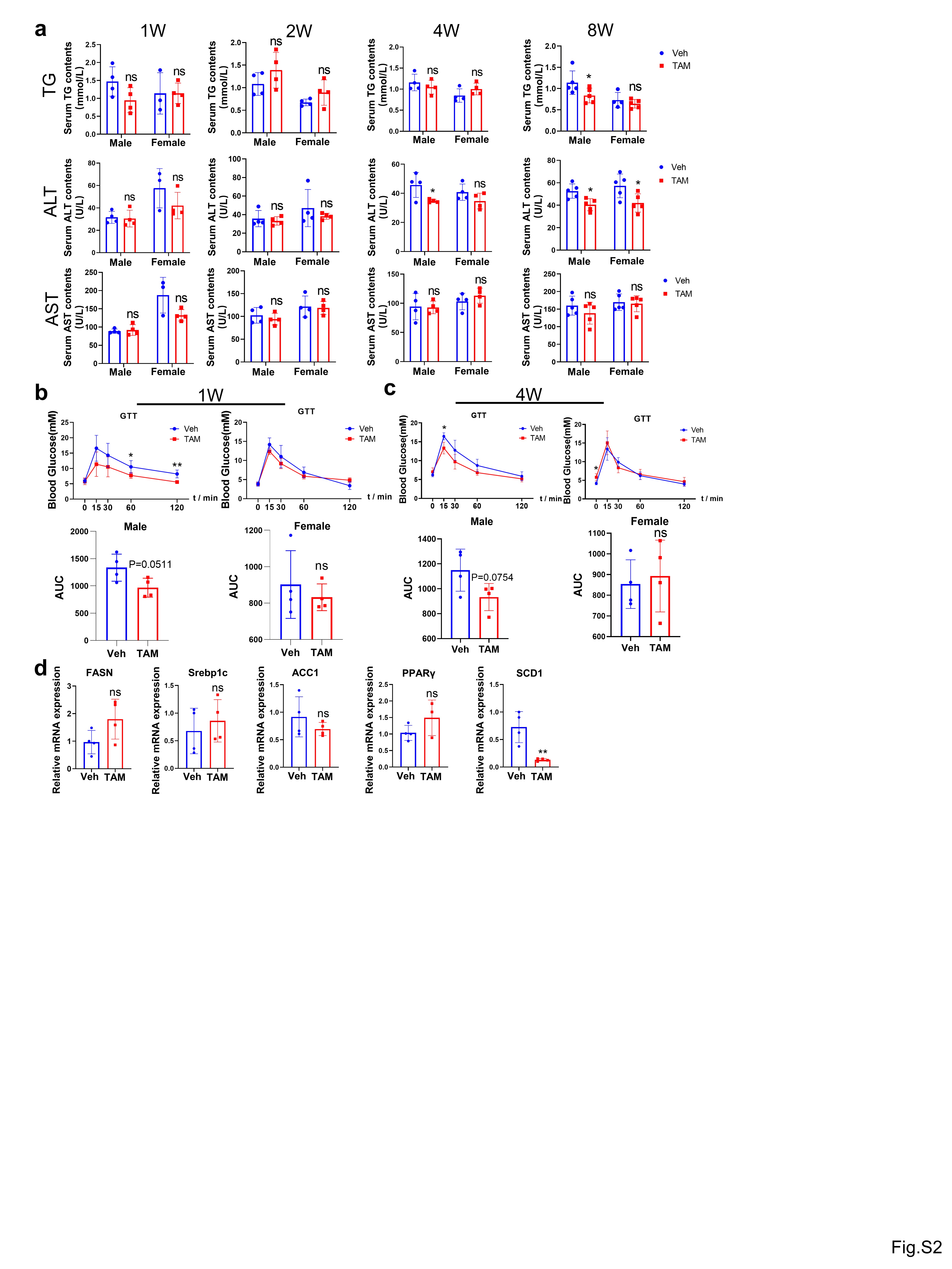


**Fig. S2** (**a**) Serum TG, ALT and AST analysis of male and female mice fed with normal diets and administrated with vehicle or 100mg/kg tamoxifen for 1, 2, 4, 8 weeks, respectively. (**b**) GTT test was performed on male and female mice administrated with tamoxifen or vehicle for a week and area under curve (AUC) was calculated and compared. (**c**) GTT test was performed on male and female mice administrated with tamoxifen or vehicle for 4 weeks and area under curve (AUC) was calculated and compared. (**d**) Total RNA was extracted from liver tissues in normal diets-fed male mice administrated with tamoxifen for 8 weeks. Lipogenic gene mRNA expression was examined by RT-qPCR. Bars = means ± SD; n=3 to 5; ns, no significance; *P < 0.05; **P < 0.01.

Figure. S3.


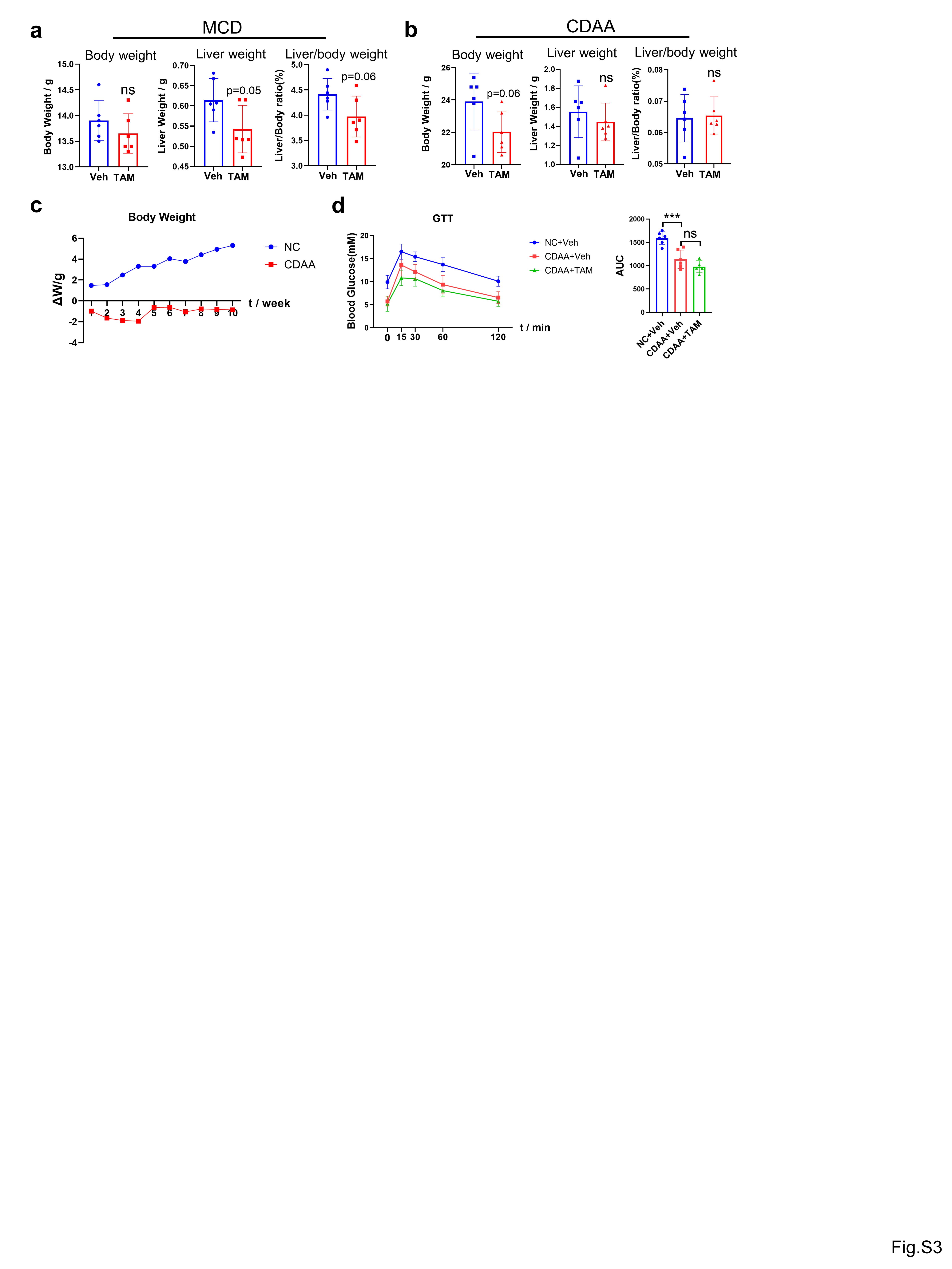


**Fig. S3** (**a**) The body weight, liver weight and liver to body ratio of male MCD diets-induced mice administrated with vehicle or 100mg/kg tamoxifen. (**b**) The body weight, liver weight and liver to body ratio of male CDAA diets-induced mice administrated with vehicle or tamoxifen. (**c**) The body weight of male mice fed with normal chow diets (NC) or CDAA diets mice and administrated with vehicle were recorded weekly and the body weight curve was manifested. (**d**) GTT test was performed on male CDAA diets-induced mice administrated with tamoxifen or vehicle and area under curve (AUC) was calculated and compared. Bars = means ± SD; n=5 to 6; ns, no significance; ***P <0.001.

Figure. S4.


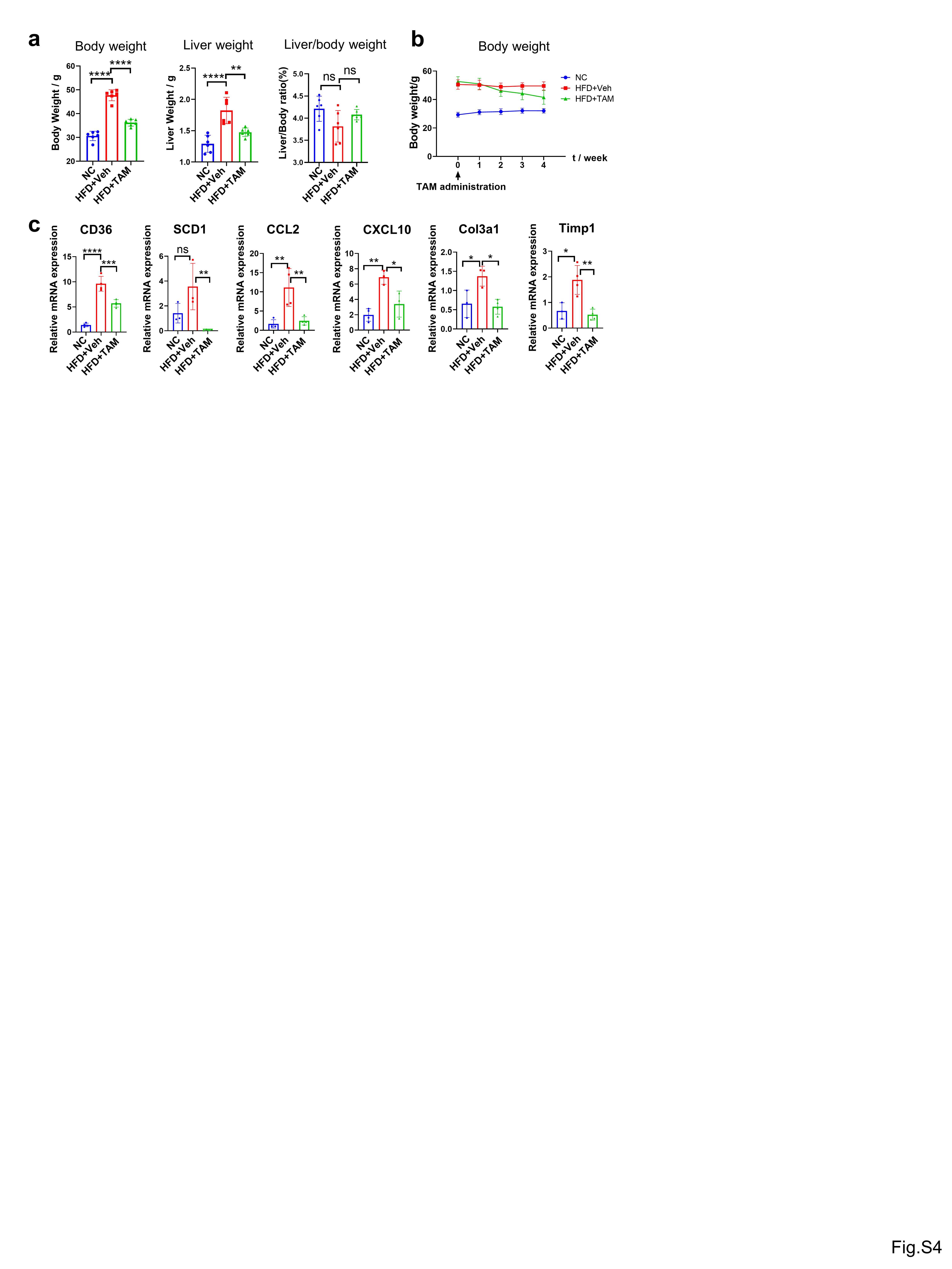


**Fig. S4** (**a**) The body weight, liver weight and liver to body ratio of male high fat diets-induced mice administrated with vehicle or 100mg/kg tamoxifen or control mice. (**b**) The body weight change after tamoxifen administration in HFD-induced NAFLD model. (**c**) RNA was extracted from liver tissues of high fat diets-induced NAFLD mice administrated with tamoxifen or vehicle or control mice and expression of lipogenesis, inflammation and fibrosis-related genes was determined by RT-qPCR with β-actin as an internal control. Bars = means ± SD; n=3 to 6; ns, no significance; *P < 0.05; **P < 0.01; ***P <0.001; ****P<0.0001.

Figure. S5.


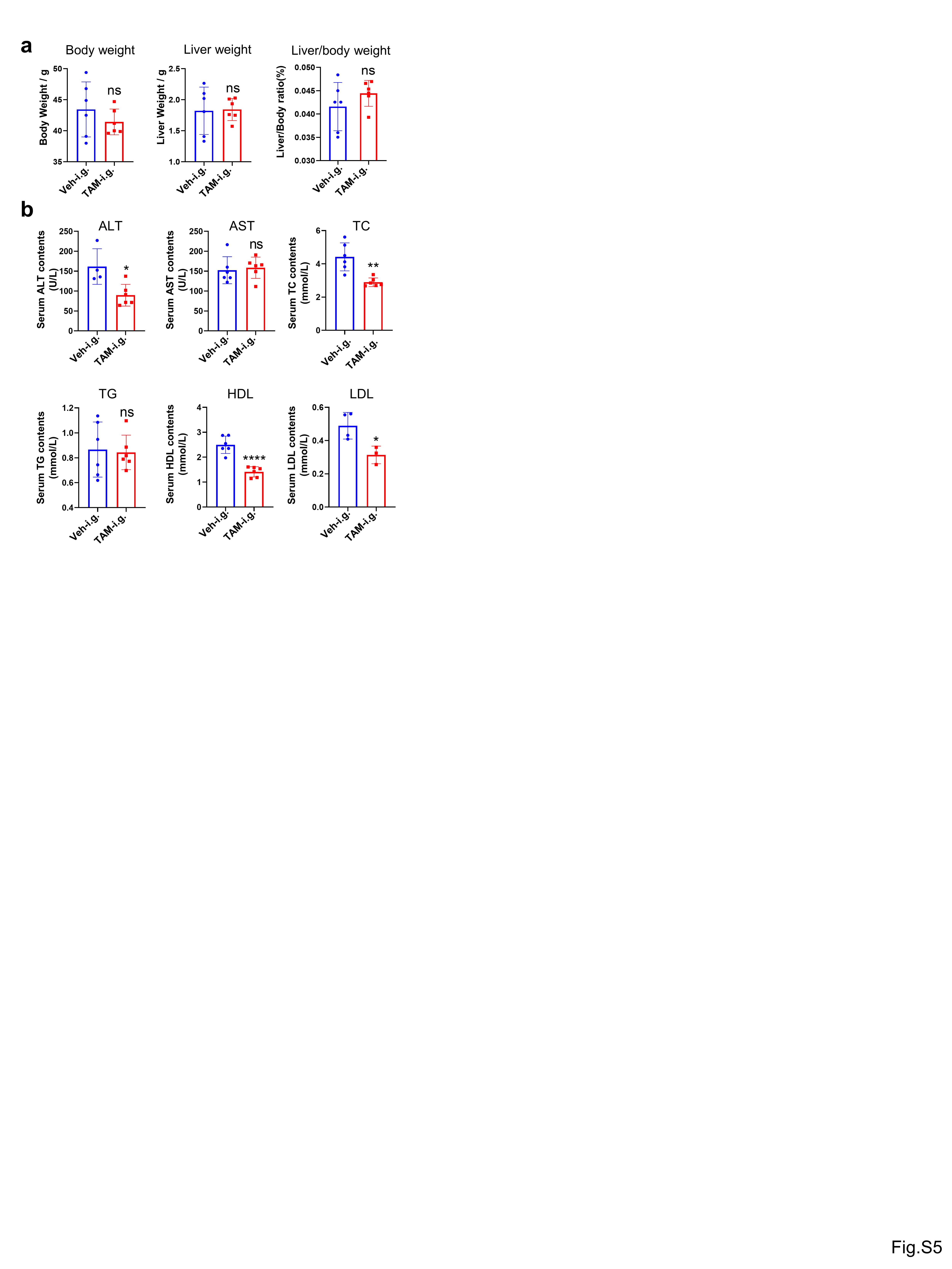


**Fig. S5** (**a**) Body weight, liver weight and liver to body weight ratio of HFD-induced NAFLD mice administrated with 100mg/kg tamoxifen or vehicle orally for 2 weeks. (**b**) Serum ALT, AST, TC, TG, HDL and LDL analysis of HFD-induced NAFLD mice administrated with tamoxifen or vehicle orally for 2 weeks. Bars = means ± SD; n=3 to 6; ns, no significance; *P < 0.05; **P < 0.01; ****P<0.0001.

Figure. S6.


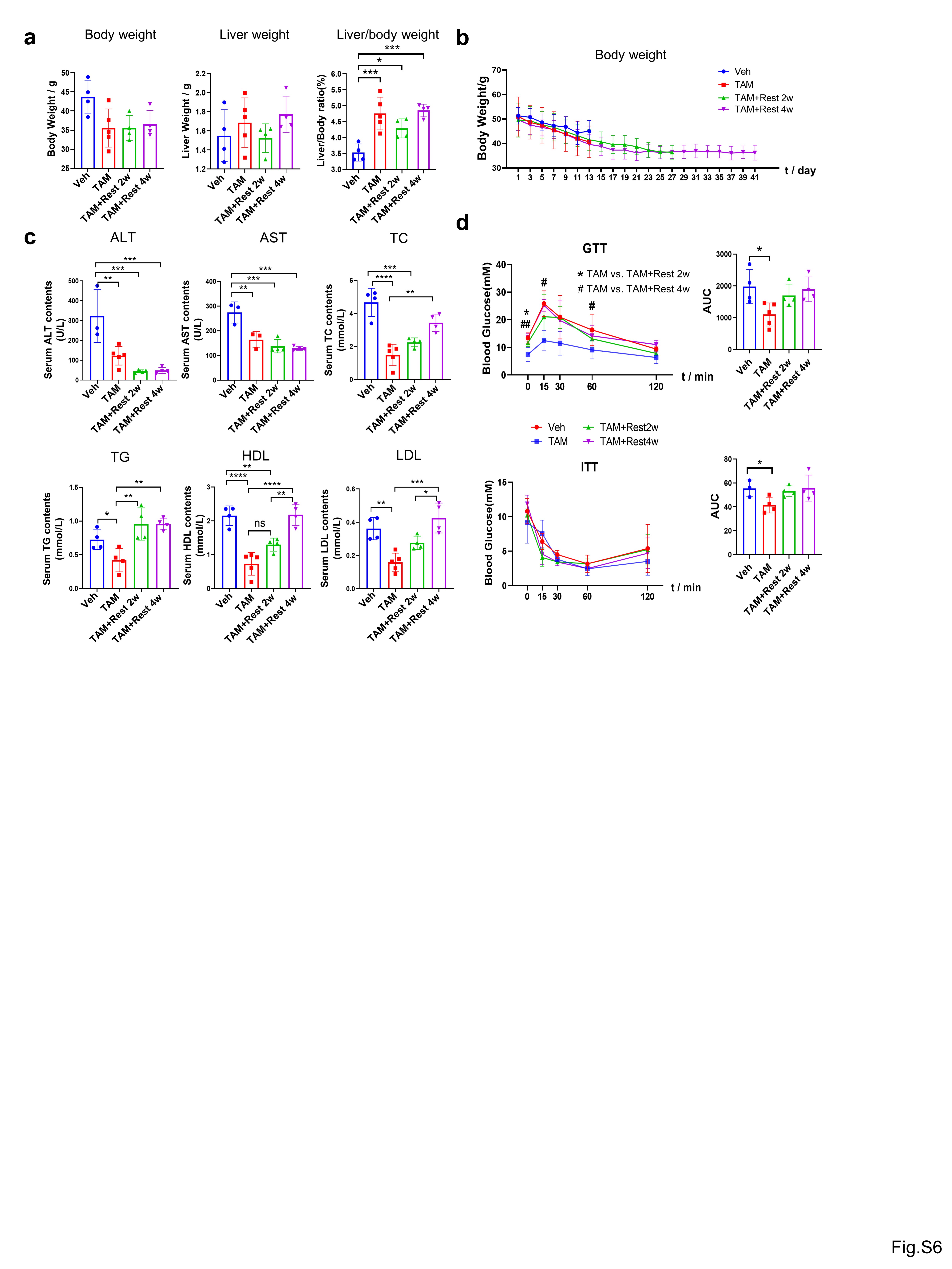


**Fig. S6** (**a**) The body weight, liver weight and liver to body weight ratio of HFD-induced NAFLD mice administrated with 100mg/kg tamoxifen or vehicle for 2 weeks and ceased treatment for 2 or 4 weeks. (**b**) The body weight curve of HFD-induced NAFLD mice administrated with tamoxifen or vehicle for 2 weeks and ceased treatment for 2 or 4 weeks. (**c**) Serum ALT, AST, TC, TG, HDL and LDL analyses of HFD-induced NAFLD mice administrated with tamoxifen or vehicle for 2 weeks and ceased treatment for 2 or 4 weeks. (**d**) GTT and ITT tests were performed on HFD-induced NAFLD mice administrated with tamoxifen or vehicle for 2 weeks and ceased treatment for 2 or 4 weeks. Bars = means ± SD; n=3 to 5; ns, no significance; *P < 0.05; **P < 0.01; ***P <0.001; ****P<0.0001.

Figure. S7.


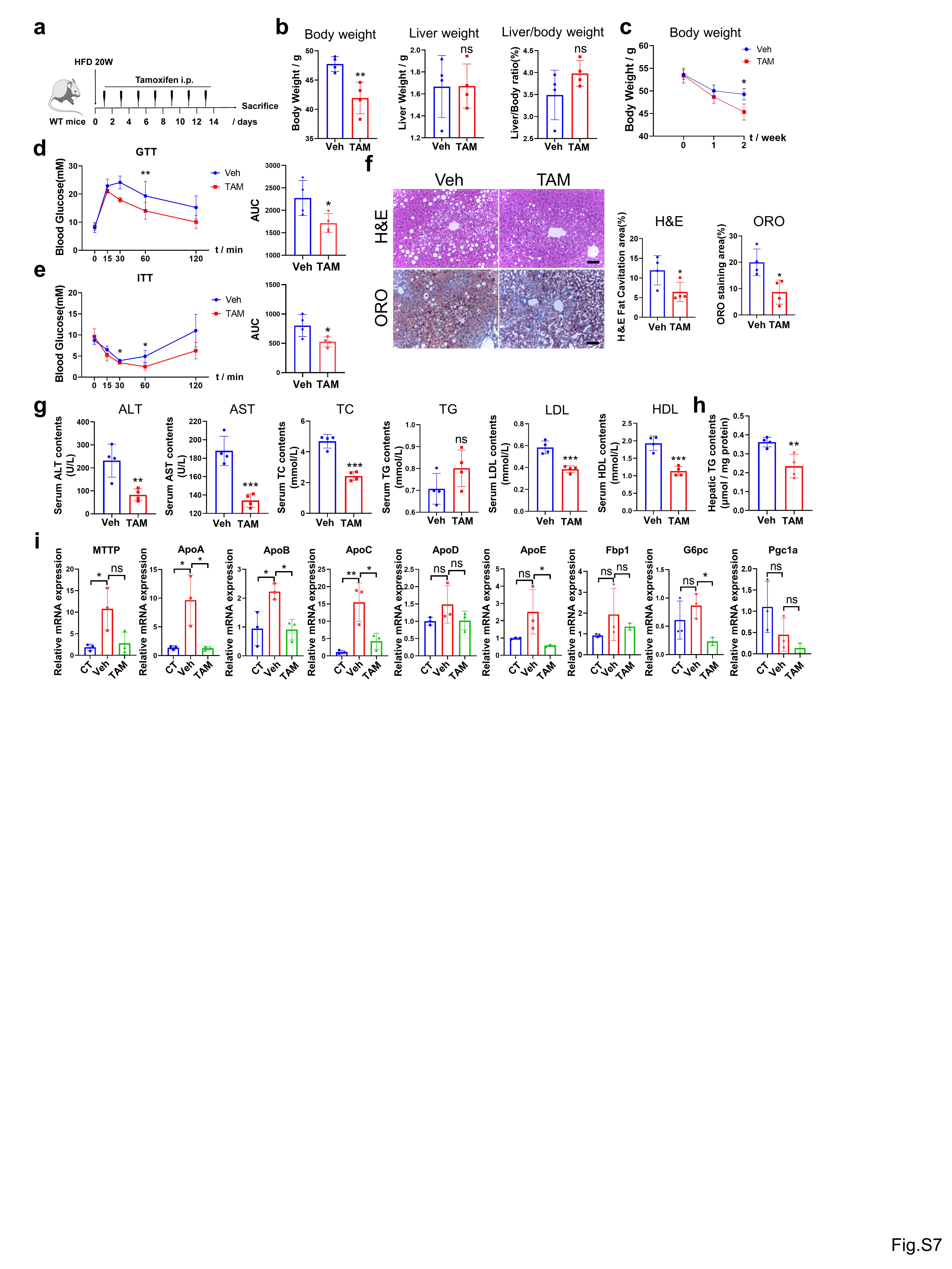


**Fig. S7** (**a**) Dosing scheme of tamoxifen on male C57BL/6 mice fed with high fat diets for 20 weeks. Dose of tamoxifen: 100 mg/kg. (**b**) The body weight, liver weight and liver to body ratio of male high fat diets-induced mice administrated with vehicle or 100mg/kg tamoxifen for 2 weeks. (**c**) The body weight change after 2-week tamoxifen administration in HFD-induced NAFLD model. (**d**) GTT test was performed on male HFD-induced mice administrated with tamoxifen or vehicle for 2 weeks and area under curve (AUC) was calculated and compared. (**e**) ITT test was performed on male HFD-induced mice administrated with tamoxifen or vehicle for 2 weeks and area under curve (AUC) was calculated and compared. (**f**) Liver sections from male HFD-induced mice administrated with tamoxifen or vehicle for 2 weeks were performed H&E and ORO staining and H&E fat cavitation area and ORO staining area was quantitatively compared. Scale bar:100μm. (**g**) Serum ALT, AST, TC, TG, HDL and LDL analysis of male HFD-induced mice administrated with tamoxifen or vehicle for 2 weeks. (**h**) Hepatic TG levels were examined and normalized by protein levels. (**i**) RNA was extracted from liver tissues and expression of TG export (MTP, ApoA, ApoB, ApoC, ApoD, ApoE) and gluconeogenesis (Fbp1, G6pc, Pgc1a)-related genes was determined by RT-qPCR with β-actin as an internal control. Bars = means ± SD; n=3 to 4; ns, no significance; *P < 0.05; **P < 0.01; ***P <0.001.

Figure. S8.


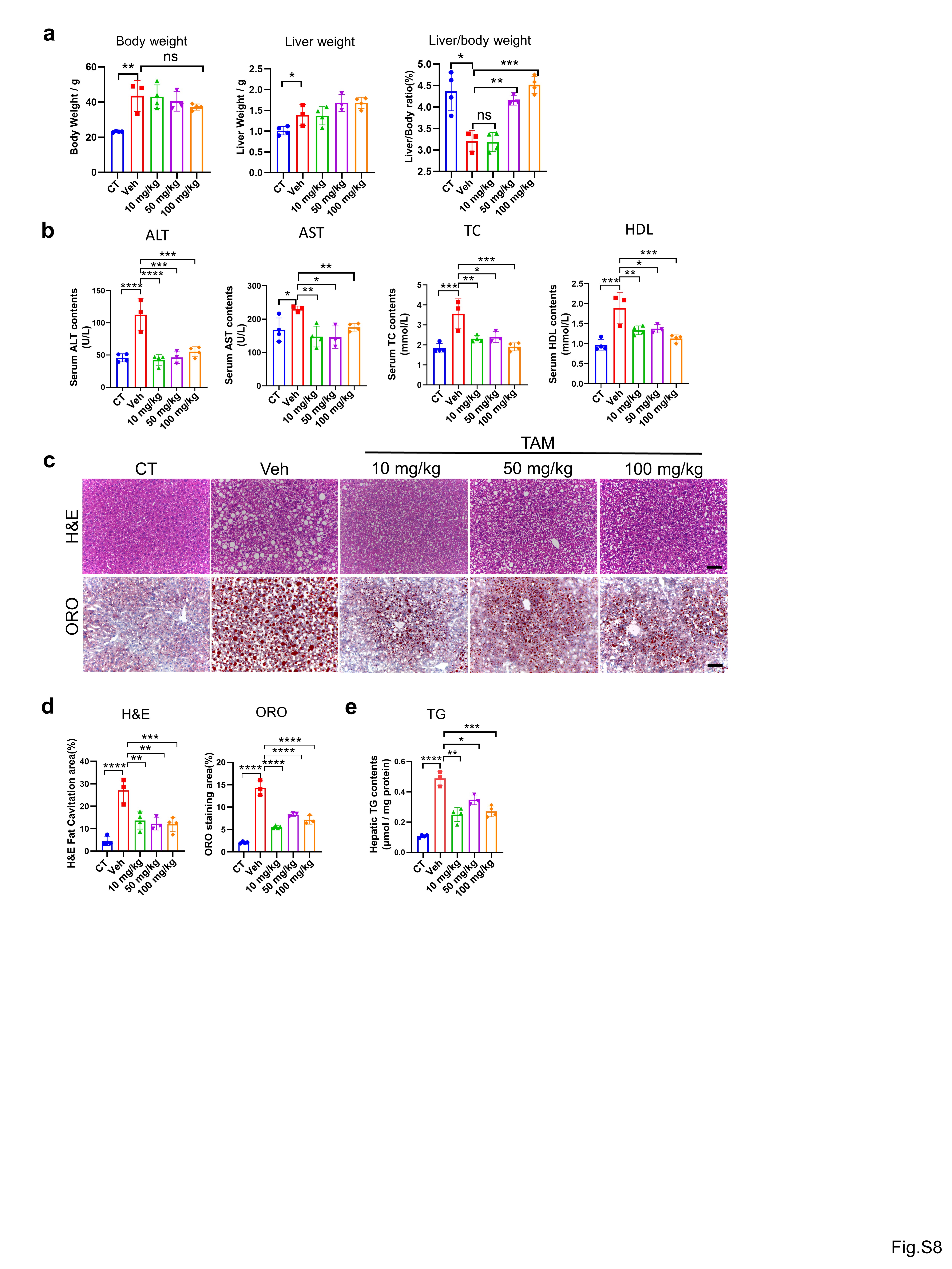


**Fig. S8** (**a**) The body weight, liver weight and liver to body weight ratio of HFD-induced mice administrated with different doses tamoxifen (10mg/kg, 50mg/kg, and 100mg/kg) or vehicle and control mice. (**b**) Serum analyses of ALT, AST, TC, and HDL-C in HFD-induced mice administrated with different doses tamoxifen (10mg/kg, 50mg/kg, and 100mg/kg) or vehicle and control mice. (**c**) H&E and ORO were performed in liver sections collected from HFD-induced mice following tamoxifen administration at different doses (10mg/kg, 50mg/kg, and 100mg/kg). (**d**) The H&E cavitation and ORO staining area were quantitively compared. (**e**) Hepatic TG levels were examined and normalized by protein levels. Scale bar:100μm. Bars = means ± SD; n=3 to 4. ns, no significance; *P < 0.05; **P < 0.01; ***P <0.001; ****P<0.0001.

Figure. S9.


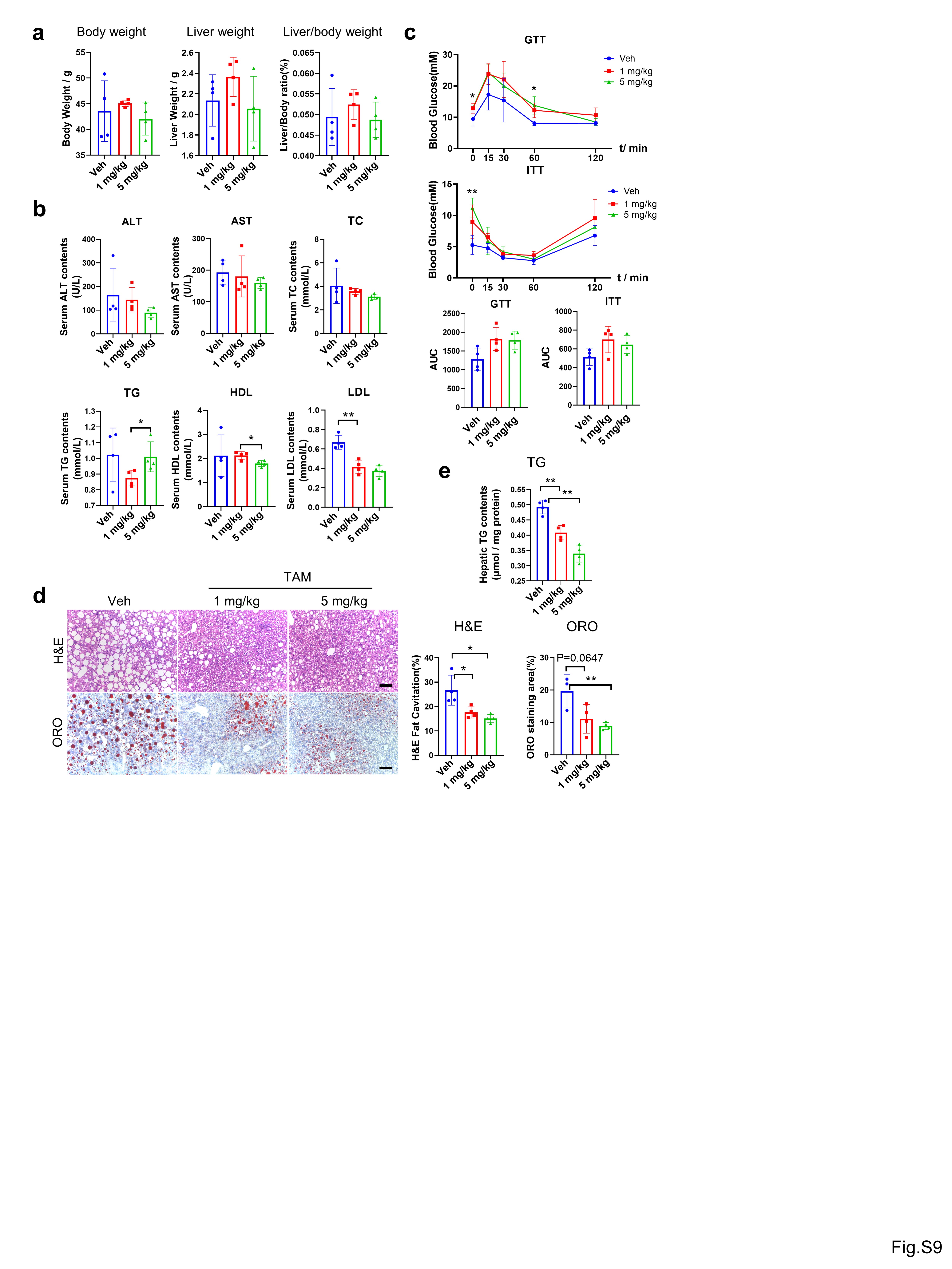


**Fig. S9** (**a**) The body weight, liver weight and liver to body weight ratio of HFD-induced mice administrated with different doses tamoxifen (1 mg/kg, 5 mg/kg) or vehicle. (**b**) Serum analyses of ALT, AST, TC, TG, HDL-C and LDL-C in HFD-induced mice administrated with different doses tamoxifen (1 mg/kg, 5 mg/kg) or vehicle. (**c**) GTT and ITT tests were performed on HFD-induced mice administrated with different doses tamoxifen (1 mg/kg, 5 mg/kg) or vehicle. (**d**) H&E and ORO were performed in liver sections collected from HFD-induced mice following tamoxifen administration at different doses (1 mg/kg, 5 mg/kg). The H&E cavitation and ORO staining area were quantitively compared. (**e**) Hepatic TG levels were examined and normalized by protein levels. Scale bar:100μm. Bars = means ± SD; n=3 to 4. *P < 0.05; **P < 0.01.

Figure. S10.


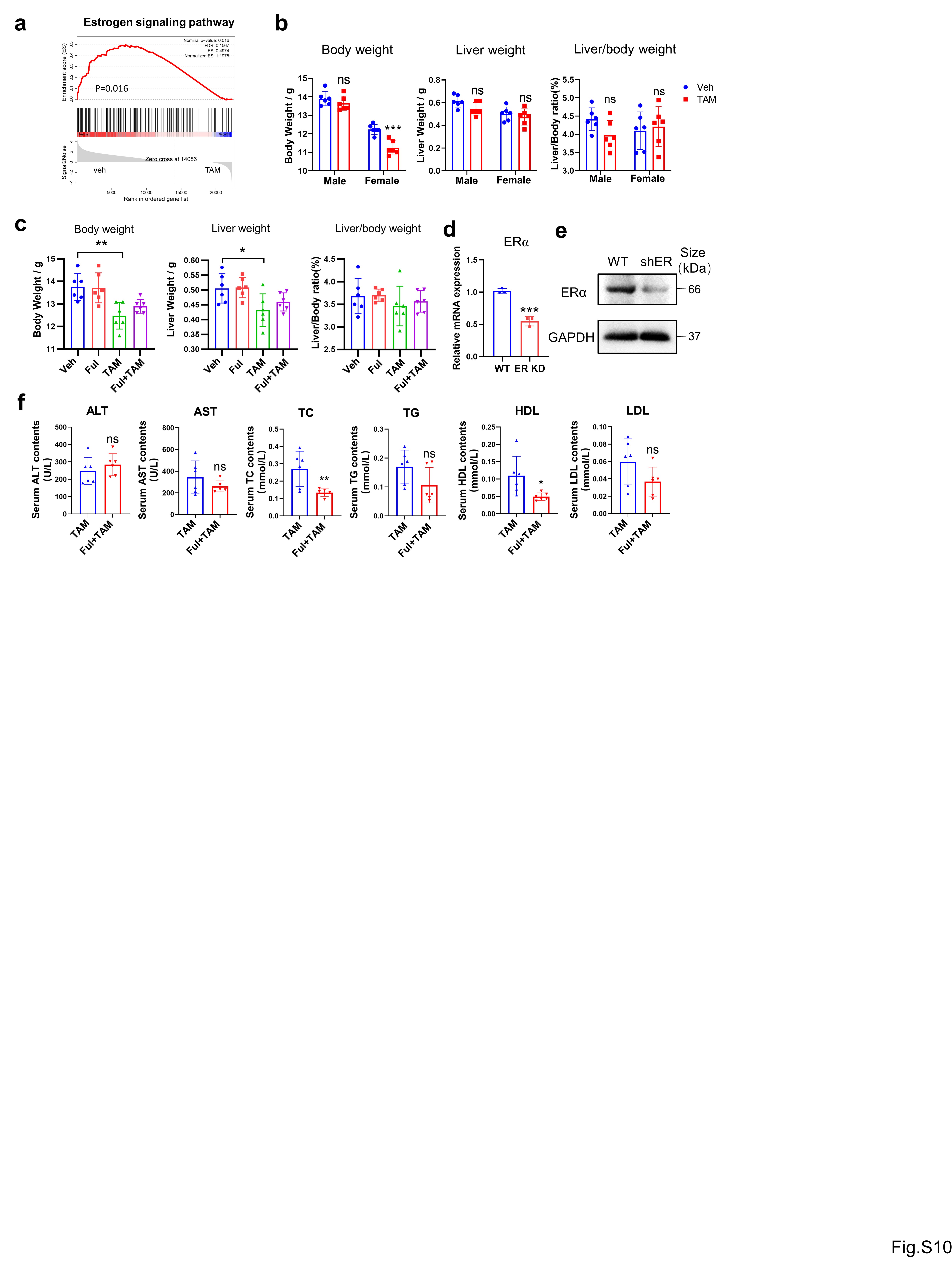


**Fig. S10** (**a**) GSEA analysis of estrogen signaling pathway. The figure was generated on Omicsmart platform (https://www.omicsmart.com/). (**b**) The body weight, liver weight and liver to body ratio of male and female mice fed with MCD diets for 6 weeks and administrated with 100mg/kg tamoxifen or vehicle intraperitoneally for 5 consecutive days. (**c**) The body weight, liver weight and liver to body ratio of male MCD diets-induced mice administrated with vehicle, fulvestrant, tamoxifen or tamoxifen + fulvestrant. (**d**) The mRNA expression of ER in WT and ER KD AML12 cells. (**e**) The protein levels of ER in WT and ER KD AML12 cells. (**f**) Serum analysis of ALT, AST, TC, TG, HDL and LDL in female MCD diets-induced mice administrated with tamoxifen or tamoxifen + fulvestrant. Bars = means ± SD; n=3 to 6; ns, no significance; *P < 0.05; **P < 0.01; ***P <0.001.

Figure. S11-1.


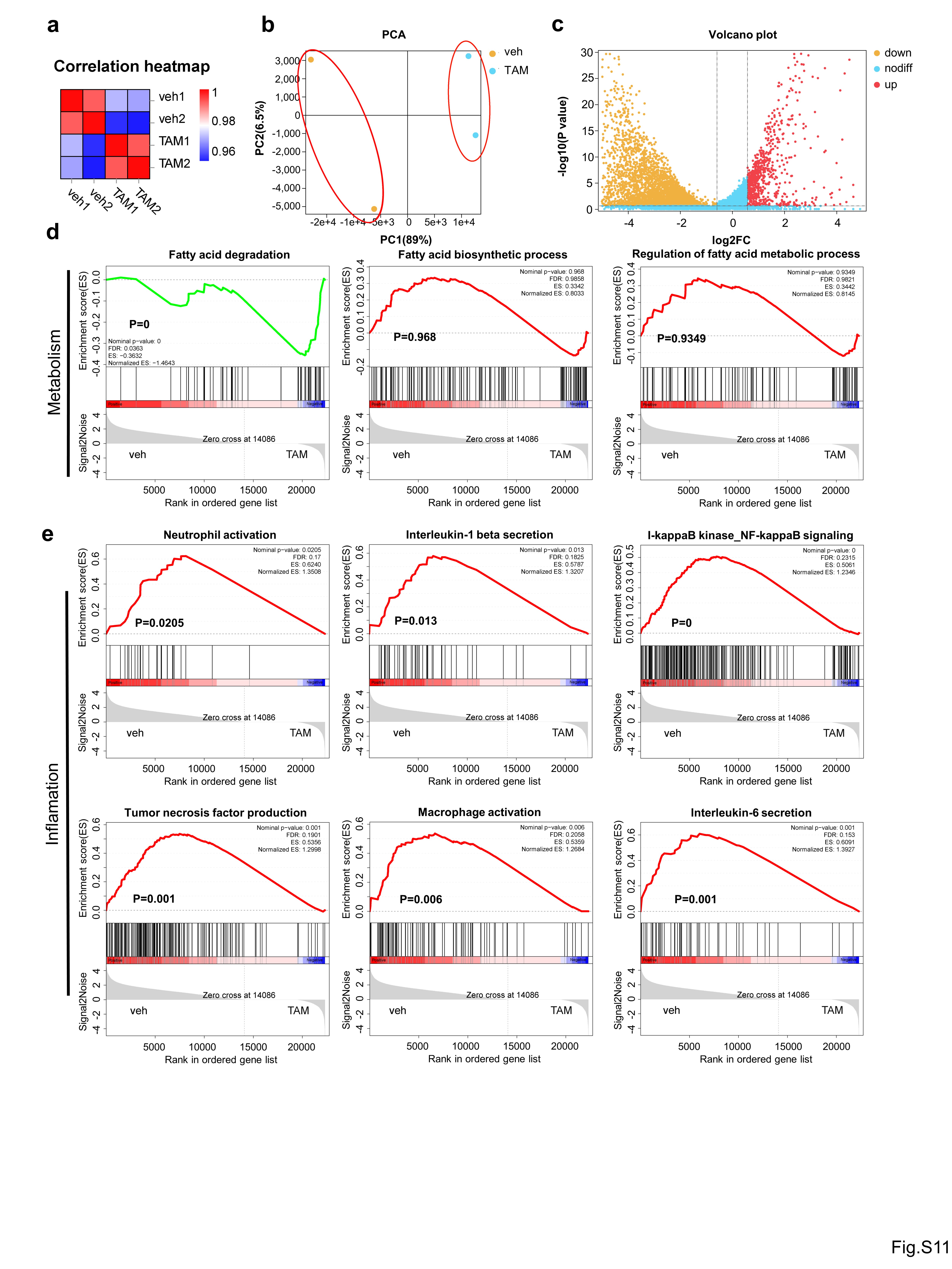


Figure. S11-2.


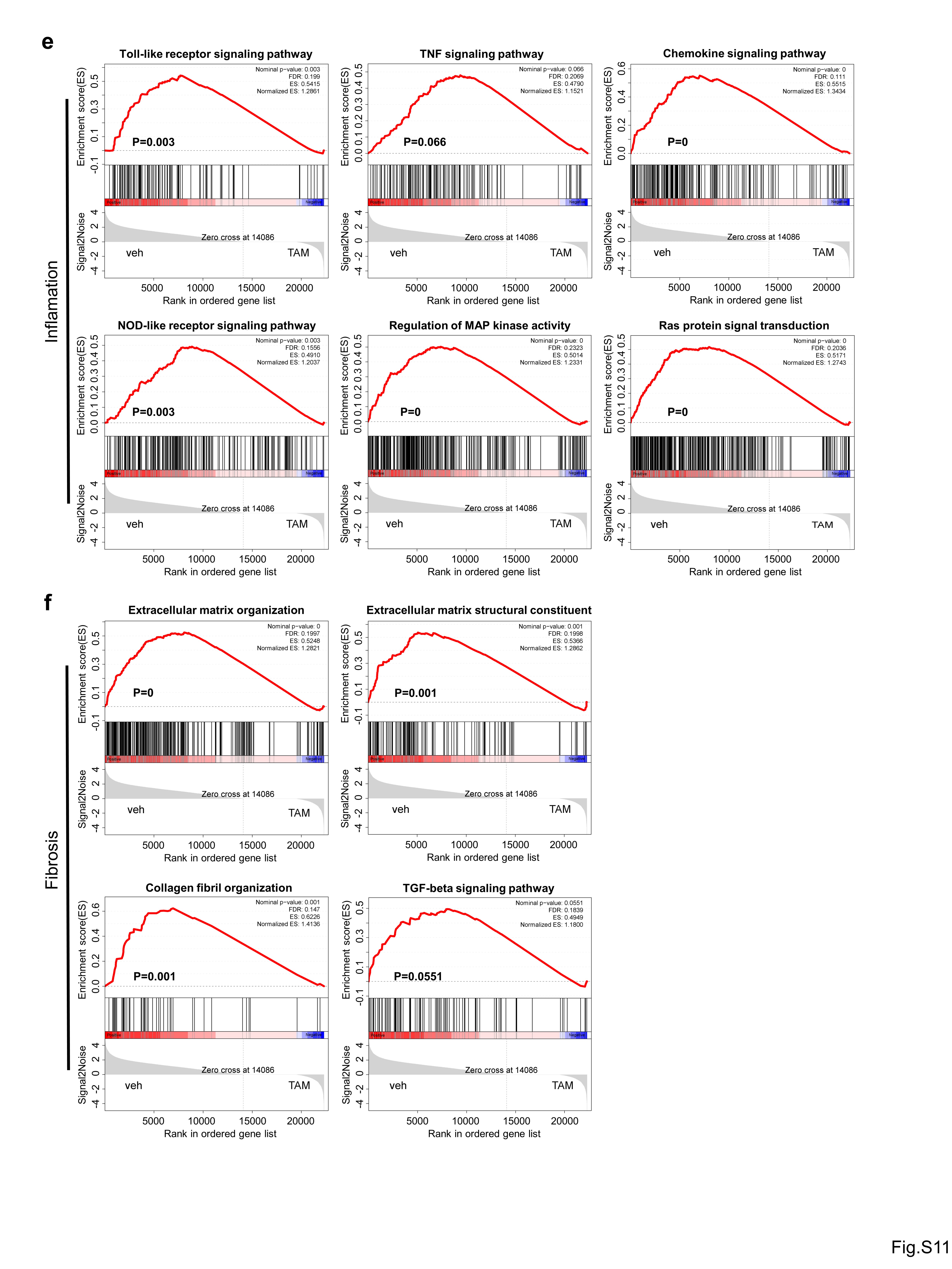


**Fig. S11** (**a**) Correlation heatmap of RNA-seq samples. (**b**) PCA analysis of RNA-seq samples. (**c**) Volcano plot of DEGs. (**d**) GSEA analysis of fatty acid degradation and biosynthesis process. (**e**) GSEA analysis of inflammation-related signaling pathway. (**f**) GSEA analysis of fibrosis-related signaling pathway. Supplementary Fig. a-f were generated on Omicsmart platform (<https://www.omicsmart.com/>).

Figure. S12.


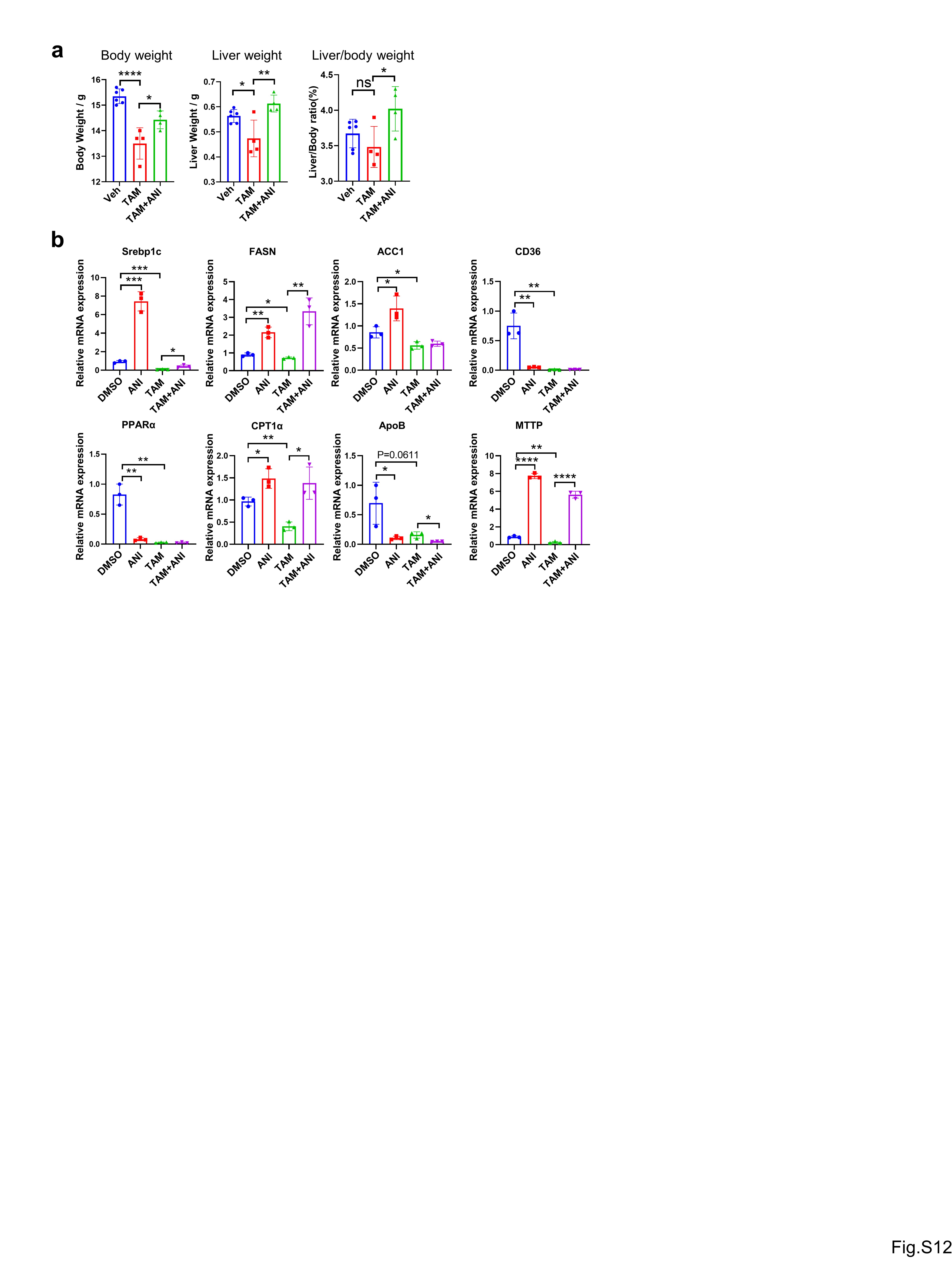


**Fig. S12** (**a**) Body weight, liver weight and liver to body ratio of MCD diets-induced mice administrated with vehicle, tamoxifen or tamoxifen + ANI, respectively. (**b**) Total RNA was extracted from AML12 cells treated with DMSO/ ANI 10 μM/ tamoxifen 40 μM / ANI 10 μM + tamoxifen 40 μM and expression of lipogenesis (Srebp1c, FASN, ACC1), fatty acids intake (CD36), fatty acid oxidation (PPARα, CPT1α) and TG export (ApoB, MTTP)-related genes was determined by RT-qPCR with β-actin as an internal control. Bars = means ± SD; n=3 to 6; ns, no significance; *P < 0.05; **P < 0.01; ***P <0.001; ****P<0.0001.

Figure. S13.


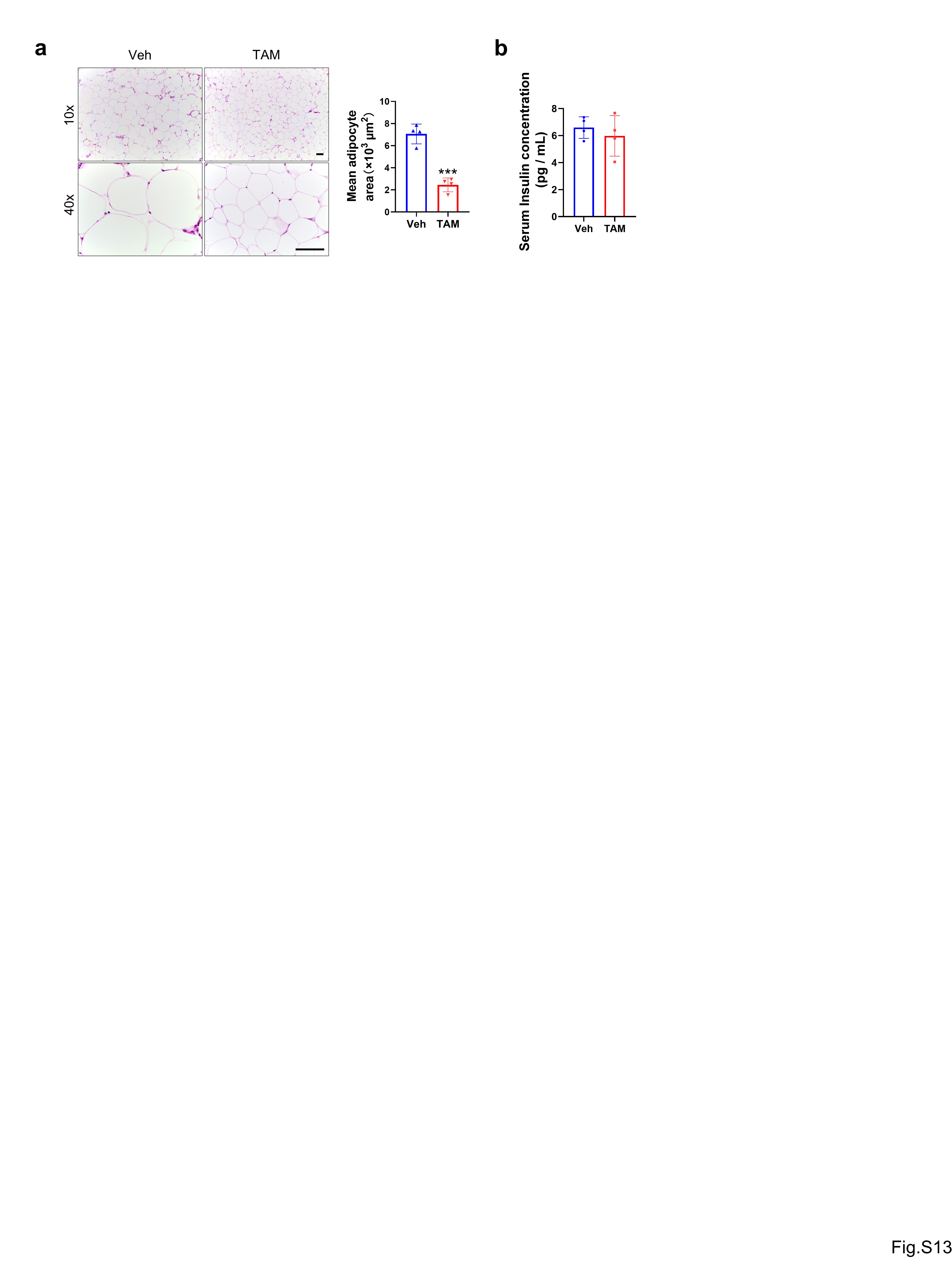


**Fig. S13** (**a**) White adipose tissues (WATs) were harvested from HFD-induced NAFLD mice administrated with 100mg/kg tamoxifen or vehicle every other day for 2 weeks and embedded in paraffin and sectioned. H&E staining was performed and mean adipocyte area was quantitatively compared. Scale bar: 100 μm. (**b**) Serum samples were collected from HFD-induced NAFLD mice administrated with tamoxifen or vehicle every other day for 2 weeks and Insulin concentration was examined using ELISA kit. Bars = means ± SD; n=4; ***P <0.001.

Table S1. Primers used for RT-qPCR

| ***Names*** | ***Species*** | ***Sequence (5′ to 3′)*** |
| --- | --- | --- |
| *Srebp1c* | *Mus musculus* | Sense: GGAGCCATGGATTGCACATT  Antisense: GGCCCGGGAAGTCACTGT |
| *SCD1* | *Mus musculus* | Sense: TCTTCCTTATCATTGCCAACACCA  Antisense: GCGTTGAGCACCAGAGTGTATCG |
| *ACC1* | *Mus musculus* | Sense: GGCCAGTGCTATGCTGAGAT  Antisense: AGGGTCAAGTGCTGCTCCA |
| *PPARα* | *Mus musculus* | Sense: TATTCGGCTGAAGCTGGTGTAC  Antisense: CTGGCATTTGTTCCGGTTCT |
| *CCL2* | *Mus musculus* | Sense: TACAAGAGGATCACCAGCAGC  Antisense: ACCTTAGGGCAGATGCAGTT |
| *α-SMA* | *Mus musculus* | Sense: CCCAGACATCAGGGAGTAATGG  Antisense: TCTATCGGATACTTCAGCGTCA |
| *CXCL10* | *Mus musculus* | Sense: ATGACGGGCCAGTGAGAATG  Antisense: ATGATCTCAACACGTGGGCA |
| *Ctgf* | *Mus musculus* | Sense: TGACCCCTGCGACCCACA  Antisense: TACACCGACCCACCGAAGACACAG |
| *IL-1β* | *Mus musculus* | Sense: CCGTGGACCTTCCAGGATGA  Antisense: GGGAACGTCACACACCAGCA |
| *CD36* | *Mus musculus* | Sense: GACTGGGACCATTGGTGATGA  Antisense: AAGGCCATCTCTACCATGCC |
| *Col1a1* | *Mus musculus* | Sense: TGCTAACGTGGTTCGTGACCGT  Antisense: ACATCTTGAGGTCGCGGCATGT |
| *Col3a1* | *Mus musculus* | Sense: ACGTAAGCACTGGTGGACAG  Antisense: CCGGCTGGAAAGAAGTCTGA |
| *Acox1* | *Mus musculus* | Sense: TCGAAGCCAGCGTTACGAG  Antisense: ATCTCCGTCTGGGCGTAGG |
| *FASN* | *Mus musculus* | Sense: CTGCGGAAACTTCAGGAAATG  Antisense: GGTTCGGAATGCTATCCAGG |
| *PPARγ* | *Mus musculus* | Sense: ATTCTGGCCCACCAACTTCGG  Antisense: TGGAAGCCTGATGCTTTATCCCCA |
| *Timp1* | *Mus musculus* | Sense: GAGACCACCTTATACCAGCGTT  Antisense: TACGCCAGGGAACCAAGAAG |
| *ER* | *Mus musculus* | Sense: CCTCCCGCCTTCTACAGGT  Antisense: CACACGGCACAGTAGCGAG |
| *CPT1α* | *Mus musculus* | Sense: AGGACCCTGAGGCATCTATT  Antisense: ATGACCTCCTGGCATTCTCC |
| *MTTP* | *Mus musculus* | Sense: ATACAAGCTCACGTACTCCACT  Antisense: TCCACAGTAACACAACGTCCA |
| *ApoA* | *Mus musculus* | Sense: GGCAGAGACTATGTGTCCCAGT  Antisense: GCTGACTAACGGTTGAACCCAG |
| *ApoB* | *Mus musculus* | Sense: AAGCACCTCCGAAAGTACGTG  Antisense: CTCCAGCTCTACCTTACAGTTGA |
| *ApoC* | *Mus musculus* | Sense: CGGAACATTGGAGAGCATACCG  Antisense: GGTCTTGGTCAAAATTTCCTTCTG |
| *ApoD* | *Mus musculus* | Sense: GGTGAAGCCAAACAGAGCAACG Antisense: CAGGAGTACACGAGGGCATAGT |
| *ApoE* | *Mus musculus* | Sense: GAACCGCTTCTGGGATTACCTG  Antisense: GCCTTTACTTCCGTCATAGTGTC |
| *Fbp1* | *Mus musculus* | Sense: TCAACTGCTTCATGCTGGAC  Antisense: GGGTCAAAGTCCTTGGCATA |
| *G6pc* | *Mus musculus* | Sense: AGCAGTTCCCTGTCACCTGT  Antisense: TGGCTTTTTCTTTCCTCGAA |
| *Pgc1α* | *Mus musculus* | Sense: CCGAGAATTCATGGAGCAAT  Antisense: TTTCTGTGGGTTTGGTGTGA |
| *FATP2* | *Mus musculus* | Sense: ACAACATTCGTGCCAAGTCTCT  Antisense: CTCCTCCACAGCTTCTTGTAGATC |
| *FATP5* | *Mus musculus* | Sense: AGCTCCTGCGGTACTTGTGT  Antisense: AAGGTCTCCCACACATCAGC |
| *β-Actin* | *Mus musculus* | Sense: GTGACGTTGACATCCGTAAAGA  Antisense: GCCGGACTCATCGTACTCC |

Table S2. List of antibodies used in this study

| ***Antibody*** | ***Catalog Number*** | ***Manufacturer*** |
| --- | --- | --- |
| *F4/80 (D4C8V) Rabbit mAb* | *#30325* | *Cell Signaling Technology* |
| *Anti -F4/80 Rabbit pAb* | *GB113373* | *Servicebio* |
| *Phospho-p38 MAPK (Thr180/Tyr182) (D3F9) Rabbit mAb* | *#4511* | *Cell Signaling Technology* |
| *p38 MAPK (D13E1) Rabbit mAb* | *#8690* | *Cell Signaling Technology* |
| *Phospho-SAPK/JNK (Thr183/Tyr185) (81E11) Rabbit mAb* | *#4668* | *Cell Signaling Technology* |
| *SAPK/JNK Antibody* | *#9252* | *Cell Signaling Technology* |
| *Phospho-p44/42 MAPK (Erk1/2) (Thr202/Tyr204) (D13.14.4E) Rabbit mAb* | *#4370* | *Cell Signaling Technology* |
| *p44/42 MAPK (Erk1/2) (137F5) Rabbit mAb* | *#4695* | *Cell Signaling Technology* |
| *ER Polyclonal antibody* | *21244-1-AP* | *Proteintech* |
| *TAK1 Polyclonal antibody* | *12330-2-AP* | *Proteintech* |
| *Phospho-TAK1 (Thr184/187) (90C7) Rabbit mAb* | *#4508* | *Cell Signaling Technology* |
| *ASK1 Polyclonal antibody* | *28201-1-AP* | *Proteintech* |
| *Phospho-ASK1 (Thr845) Antibody* | *#3765* | *Cell Signaling Technology* |
| *GAPDH Polyclonal antibody* | *10494-1-AP* | *Proteintech* |
| *Goat anti Rabbit Cy3* | *111-165-003* | *Jackson Immuno Research* |
| *Goat anti-Rabbit-HRP* | *G1215-200T* | *Servicebio* |
